# Nucleophosmin3 carried by small extracellular vesicles contribute to white adipose tissue browning

**DOI:** 10.1186/s12951-022-01381-1

**Published:** 2022-03-28

**Authors:** Yan Zhang, Mei Yu, Jia Dong, Yue Wu, Weidong Tian

**Affiliations:** 1 State Key Laboratory of Oral Disease & National Clinical Research Center for Oral Diseases, West China School of Stomatology, Sichuan University, Chengdu, China; 2 National Engineering Laboratory for Oral Regenerative Medicine, West China School of Stomatology, Sichuan University, Chengdu, China; 3 Engineering Research Center of Oral Translational Medicine, Ministry of Education, Sichuan University, Chengdu, China; 4 Department of Oral and Maxillofacial Surgery, West China Hospital of Stomatology, Sichuan University, Chengdu, China; 5 Department of Oral and Maxillofacial Surgery, Tianjin Stomatological Hospital, School of Medicine, Nankai University, Tianjin, China

**Keywords:** NPM3, White adipose tissue browning, Small extracellular vesicles, Obesity, Batokine

## Abstract

**Background:**

Browning of white adipose tissue (WAT) is a particularly appealing target for therapeutics in the treatment of obesity and related metabolic diseases. Although small extracellular vesicles (sEVs) released from adipose tissue (sEVs-AT) have emerged as novel player that regulate systemic metabolism by connecting different organs, the role of specific contents in sEVs-AT played in WAT browning has not been clarified.

**Results:**

We revealed Nucleophosmin3 (NPM3), which was mainly transferred by sEVs derived from brown adipose tissue (sEVs-BAT), was served as a batokine that could induce WAT browning by regulating the stability of PRDM16 mRNA. sEVs-BAT enhanced the expressions of browning related genes in 3T3-L1 preadipocytes and WAT while knocking down of NPM3 in BAT impaired sEVs-BAT mediated WAT browning and weight loss in obesity.

**Conclusion:**

These data provided new insight into the role of NPM3 in regulating the browning of WAT. Our study indicated that a supplement of sEVs-BAT might represent a promising therapeutic strategy to promote thermogenesis and energy expenditure in the future.

**Graphical Abstract:**

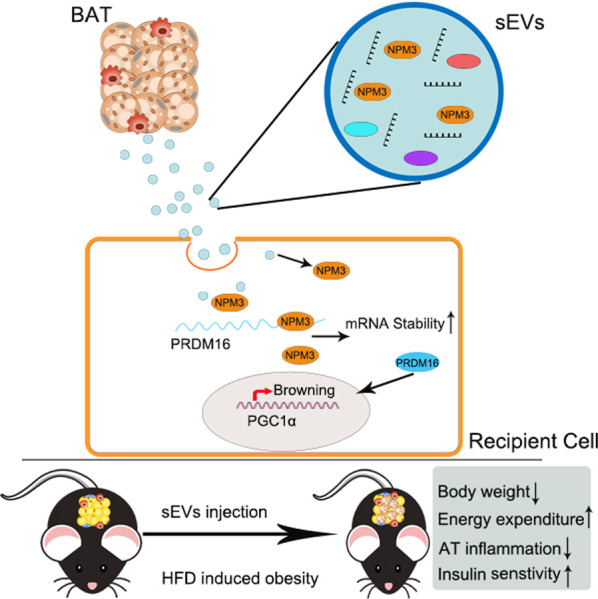

**Supplementary Information:**

The online version contains supplementary material available at 10.1186/s12951-022-01381-1.

## Introduction

Adipose tissue is commonly categorized into three types including white adipose tissue (WAT), brown adipose tissue (BAT) and beige adipose tissue [1]. WAT stores energy and provides energy during fasting to maintain energy homeostasis [2, 3]. In contrast to WAT, BAT is served as a thermogenic tissue and expends energy by consuming glucose and fatty acids [4]. Brown adipocytes are distinct from the white adipocytes with a structure of multilocular lipid droplets, and a large amount of mitochondria in the cytoplasm [5]. In fact, BAT is the main lipid clearance organ and has the highest fatty acid oxidation rate under cold exposure [6]. Thus, BAT activation might be a new approach to treat obesity and obesity-induced disorders. Recently, several studies have shown that activation or transplantation of BAT could promote caloric consumption, reduce obesity and alleviate diabetes [7–10]. Moreover, WAT attains thermogenic properties following cold exposure or β-adrenergic stimulation and is termed as beige adipose tissue. These processes are also referred to as WAT browning [11]. Due to the amount of BAT is low and decreased in adults and correlates inversely with BMI and obesity, WAT browning has gained a lot of attention in the context of the obesity epidemic worldwide [12, 13].

The browning induction of white adipose tissue in response to physiological and environmental changes (cold challenge, fasting, exercise et al.) was regulated by the release of endocrine and paracrine factors from metabolically active organs (BAT, beige AT, muscle, heart et al.). Recent studies have proved that several adipokines [including fibroblast growth factor 21 (FGF21), Slit2‑C, insulin-like growth factor 1 (IGF1)] secreted by BAT or beige AT could induce the browning of WAT and promote metabolic processes [14–16]. This indicated that adipokines acted as regulators of WAT browning by mediating the crosstalk between different adipose tissues. Recently, small extracellular vesicles (sEVs) have emerged as a new intercellular communication system between adipose tissue and distal organs for transporting adipokines [17–22]. Although the previous study reported that BAT derived sEVs (sEVs-BAT) promoted energy expenditure through regulating oxygen consumption in recipient cells and several sEVs adipokines (e.g., miR-99b, eNAMPT) have been reported to contribute to the regulation of cell metabolism in the adipose tissue or distal organs, the role of adipokines in sEVs played in WAT browning is still elusive [18, 23–25].

Our previous study indicated that nucleophosmin3 (NPM3) was enriched in sEVs derived from adipose tissue and might serve as an undefined adipokine [26]. The nucleophosmin/nucleoplasmin (NPM) family was abundantly expressed throughout the animal kingdom and can be subdivided into four groups based on the protein sequences: NPM1, NPM2, NPM3 and invertebrate NPM proteins [27]. The structure of NPM is mainly divided into three distinct regions: the N-terminal domain (protein binding), the acidic domains (histone binding), and the C-terminal nucleic acid-binding domain. Recently, NPM1 has attracted much attention and shuttles between the nucleus and the cytoplasm to regulate chromatin remodeling, genome stability, ribosome biogenesis, DNA duplication and mRNA transcription [28]. However, the functions of NPM3 have largely unclarified and although NPM3 were abundantly expressed in the adipose tissue, it is still unknown whether NPM3 plays a role in adipose development or served as a putative adipokine. In this study, we identified the critical role of NPM3 in the regulation of WAT browning and amelioration of HFD (high-fat diet) induced obesity, the results implicated a potential anti-obesity strategy in the future.

## Results

### Characterization of NPM3

**Fig. 1:**
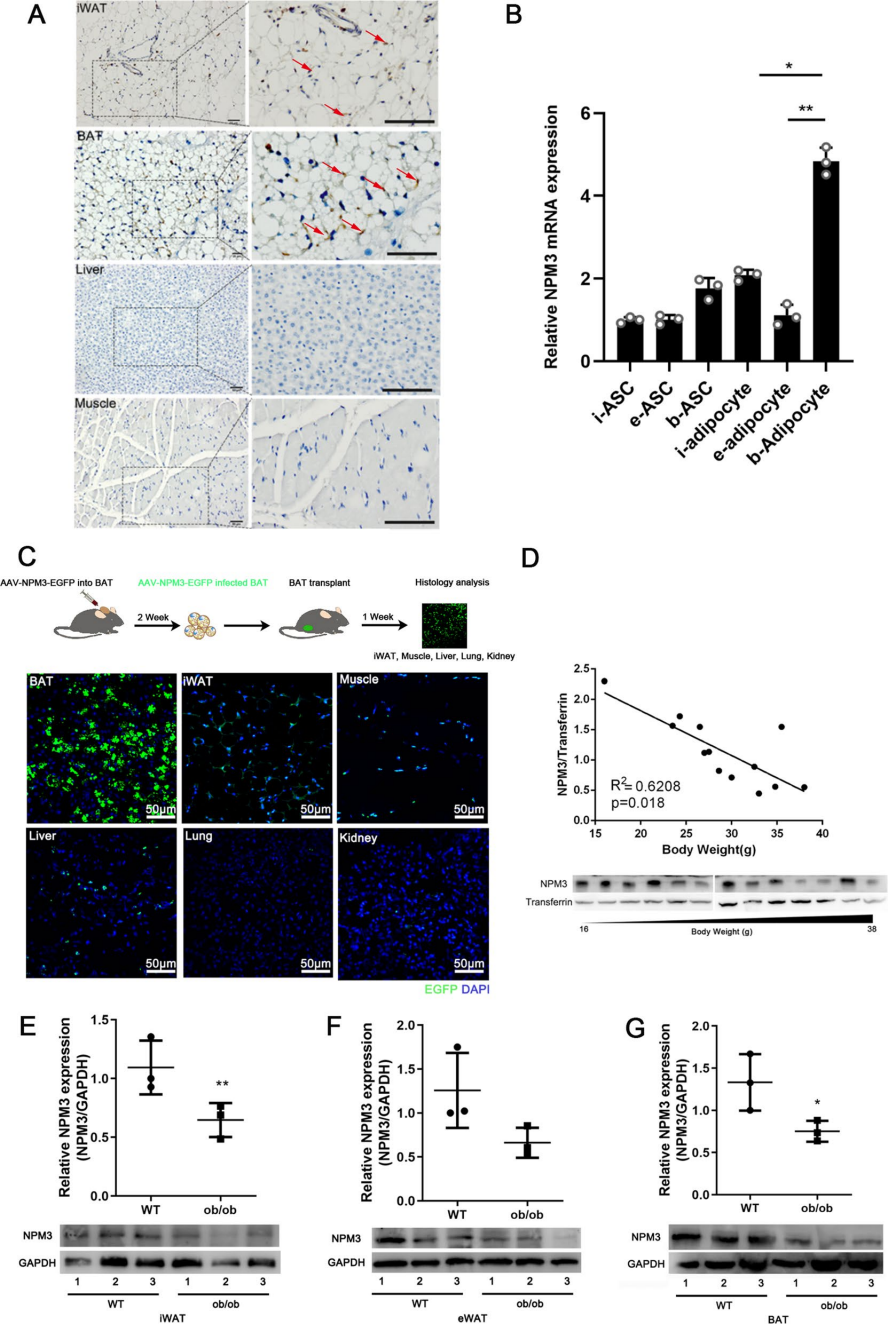
NPM3 was enriched in BAT and decreased in obesity. **A** IHC analysis of NPM3 expression in different tissues (WAT, BAT, Liver and Muscle) from 8-week-old male mice, the boxed region was shown at higher magnification at right (scale bar = 50 μm), the expression of NPM3 was pointed by red arrows; **B** qRT-PCR analysis of relative NPM3 mRNA expressions in ASCs and adipocytes from inguinal adipose tissue (iWAT), epididymal adipose tissue (eWAT) and BAT, data were represented as mean ± SEM and analyzed by one-way ANOVA followed by Tukey’s test, *P < 0.05, **P < 0.01; **C** AAV-NPM3-EGFP infected BAT was transplanted into the inguinal site of the 8-week old mice and the expressions of EGFP were detected in the iWAT, muscle, liver, lung and kidney after 1 week; **D** Relationship of plasma NPM3 levels of mice across different bodyweight groups (n = 13) (Linear regression analysis); NPM3 protein expression (n = 3) in iWAT (**E**), eWAT (**F**) and BAT (**G**) of 3-month-old male ob/ob mice and lean mice, data were represented as mean ± SEM and analyzed by student t test, *p < 0.05

To verify whether NPM3 carried the characters as an adipokine, tissue distribution of NPM3 was firstly detected in mice, the results showed that NPM3 was most abundantly expressed in BAT, followed by inguinal adipose tissue (iWAT) (Fig. 1A). We also detected the expressions of NPM3 in adipocytes and stem cells (ASCs) derived from different types of adipose, the results showed that NPM3 was specifically enriched in brown adipocytes (Fig. 1B). Moreover, we noted that NPM3 was upregulated in the plasma and iWAT when mice were under cold exposure, this indicated that browning of adipose tissue increased expressions of NPM3 (Additional file 1: Fig. S1). To further validate whether BAT derived NPM3 could be transferred to other tissues, BAT with EGFP labeled NPM3 (NPM3-EGFP AAV infected BAT) was transplanted into the inguinal site of the mice. We found that EGFP could be detected not only in the adjacent iWAT and muscle but also the distant organs such as liver, lung and spleen. This indicated that NPM3 could be delivered to distant tissue through circulation. (Fig. 1C). Moreover, there was a negative linear correlation of plasma NPM3 with body weight (Fig. 1D). In obesity, we noted that the expression level of NPM3 was significantly decreased in the iWAT (Fig. 1E), eWAT (Fig. 1F) and BAT (Fig. 1G) of the ob/ob mice compared to the lean mice. These data strongly suggested that NPM3 possessed the characteristic as an adipokine.

### NPM3 promotes WAT browning through stabilizing PRDM16 mRNA

**Fig. 2:**
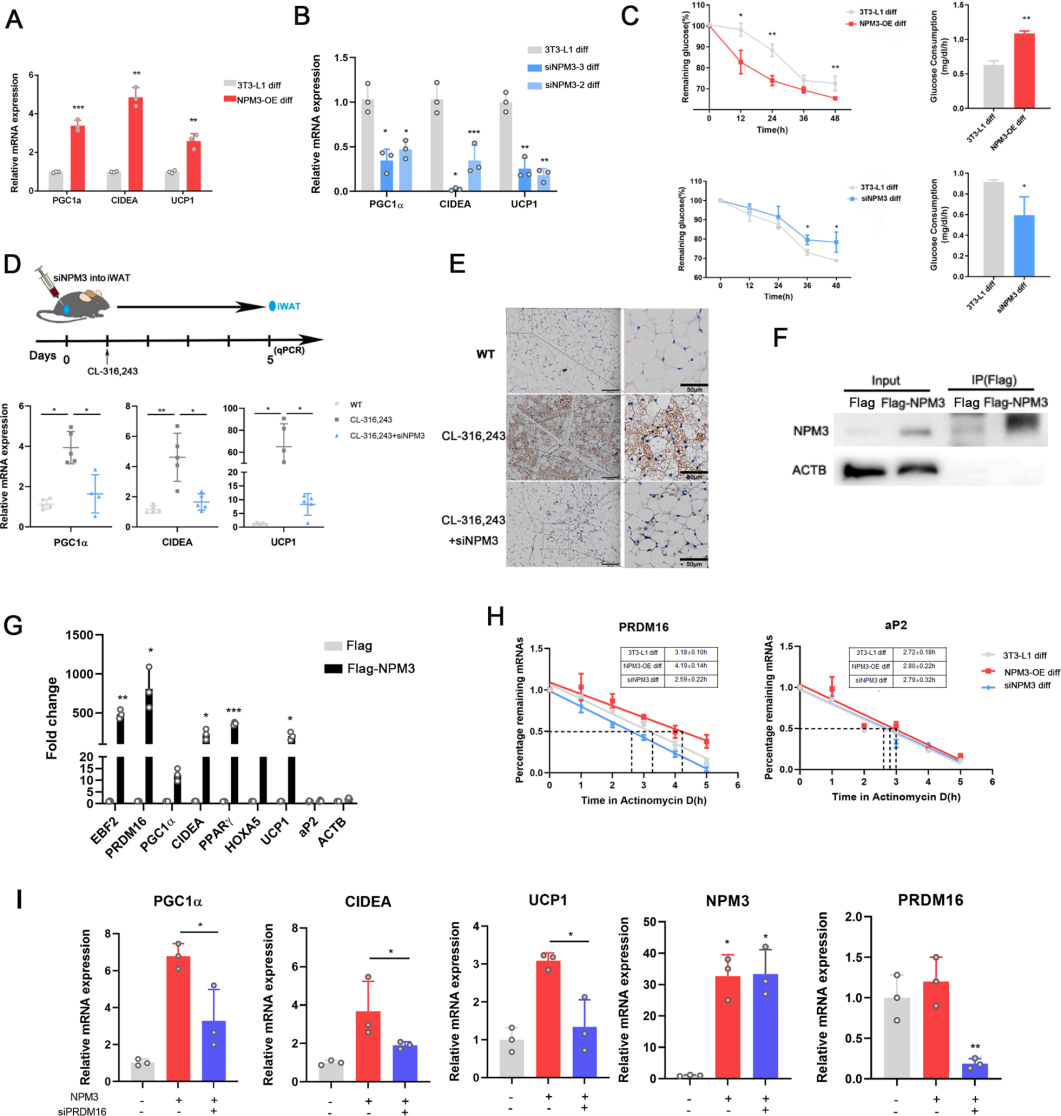
NPM3 promoted WAT browning by stabilizing PRDM16 mRNA. Relative mRNA expressions of PGC-1α, CIDEA and UCP1 after browning induction in 3T3-L1 preadipocytes (3T3-L1 diff) transfected with or without NPM3 overexpression plasmids (NPM3-OE diff) (**A**) or siRNAs (siNPM3 diff) (**B**) (n = 3), data were represented as mean ± SEM and analyzed by student t test; *P < 0.05, **P < 0.01, ***P < 0.001; **C** Glucose consumption in 3T3-L1 preadipocytes after transfected with NPM3 overexpressed plasmids or siNPM3 (n = 3), data were represented as mean ± SEM and analyzed by student t test, *P < 0.05, **P < 0.01; **D** Relative mRNA expressions of PGC-1α, CIDEA and UCP1 in iWAT with or without CL-316,243/siNPM3 treatment (n = 5), data were represented as mean ± SEM and analyzed by one-way ANOVA followed by Tukey’s test, *P < 0.05, **P < 0.01, ***P < 0.001; **E** Representative HE and UCP1 immunohistochemical staining in iWAT with or without CL-316,243/siNPM3 treatment, scale bar = 100 μm, the boxed region was shown at higher magnification at right (scale bar = 50 μm); **F** Western blot to confirm immunoprecipitation (IP) of NPM3; 10% IP cell lysate was used as the input; **G** Enrichment of selected targets was compared based on NPM3 IP vs. IgG control (n = 3), data were represented as mean ± SEM and analyzed by student t test, *P < 0.05, ***P < 0.001; **H** qRT-PCR was used to determine the remaining PRDM16 mRNA level compared with the starting time after Actinomycin D treatment, aP2 mRNA was used as a control (n = 3), data were represented as mean ± SEM and analyzed by student t test, *P < 0.05, ***P < 0.001; **I** Thermogenic marker genes were examined in 3T3-L1 cells transfected with or without NPM3 or siPRDM16 followed by browning induction (n = 3), data were represented as mean ± SEM and analyzed by student t test, *P < 0.05, ***P < 0.001

To further investigate the effects of NPM3 on WAT browning, NPM3 was upregulated or downregulated in 3T3-L1 preadipocytes (Additional file 1: Fig. S2), then go further for browning induction. Firstly, we confirmed that 3T3-L1 cells could be induced into brown adipocytes successfully (Additional file 1: Fig. S3). The expressions of thermogenic genes were upregulated by NPM3 overexpression (Fig. 2A) while reduced by NPM3 repression (Fig. 2B). The effect of NPM3 on the browning differentiation was further verified in the primary adipose stems cells derived from BAT and WAT (Additional file 1: Fig. S4). Considering that the increased numbers of brown adipocytes contributed to the high glucose consumption, the glucose consumption of 3T3-L1 preadipocytes was also detected. The upregulation of glucose consumption in the NPM3 overexpression group implied that the browning process was enhanced while this effect was impaired when NPM3 was silenced. (Fig. 2C). To investigate the role of NPM3 on WAT browning in vivo, iWAT NPM3 in CL-316,243 induced mice was knocked down by local injection of siNPM3 (Fig. 2D). We noted that CL-316,243 promoted expressions of all thermogenic markers while the elevated expressions of thermogenic markers significantly decreased when NPM3 was downregulated (Fig. 2D). Histologically, knockdown of NPM3 caused a substantial decrease in the number of multilocular adipocytes within the iWAT, along with a decrease in UCP1 positive cells (Fig. 2E), which implied that knockdown of NPM3 impaired WAT browning. NPM3 was also knocked down in the mice under cold exposure (Additional file 1: Fig. S5A), the elevated expressions of browning-related marker genes (Additional file 1: Fig. S5B) and UCP1 protein (Additional file 1: Fig. S5C) induced by cold exposure were both impaired by siNPM3 treatment. These results indicated that NPM3 was involved in the regulation of WAT browning both in vitro and in vivo.

Since NPM3 has been reported as a putative RNA binding protein (RBP) [29, 30], the cellular localization of NPM3 during browning induction was detected. During browning induction, the expressions of NPM3 in the nuclear decreased while the cytoplasmic NPM3 increased (Additional file 1: Fig. S6). This implied that NPM3 was likely involved in the posttranscriptional RNA networks with browning related gene. Thermogenic mRNAs were further tested with RNA binding protein immunoprecipitation (RIP) assay to seek the binding targets of NPM3. Due to the lack of suitable commercial anti-mouse NPM3 antibody, Flag-NPM3 were expressed in 3T3-L1 preadipocytes for RIP studies. We noted NPM3 could be detected using flag antibody while ACTB could not be detected (Fig. 2F), which indicated that Flag-NPM3 could be used for further RIP assay. The results of RIP assay indicated that EBF2, PRDM16, PPARγ, CIDEA were enriched in the Flag-NPM3-IP sample, while aP2 mRNA was not detected, indicating that aP2 did not interact with NPM3, could be used as a negative control (Fig. 2G). Among the detected thermogenic targets of NPM3, PRDM16 was the most abundant one. Therefore, we further studied how NPM3 affected PRDM16 mRNA. Given that RBPs could regulate mRNA stability, we reasoned that NPM3 might enhance the stability of PRDM16 mRNA to increase PRDM16 mRNA abundance. Compared with the non-binding gene aP2, NPM3 overexpression significantly prolonged PRDM16 mRNA half-life (Fig. 2H). To further validate the involvement of PRDM16 in the NPM3-mediated WAT browning, we knocked down PRDM16 in 3T3-L1 preadipocytes with or without the NPM3 overexpression. Knockdown of PRDM16 impaired expressions of thermogenic genes resulting from NPM3 overexpression (Fig. 2I). These results indicated that NPM3 promoted WAT browning through stabilizing PRDM16 mRNA.

### NPM3 was localized to sEVs

**Fig. 3:**
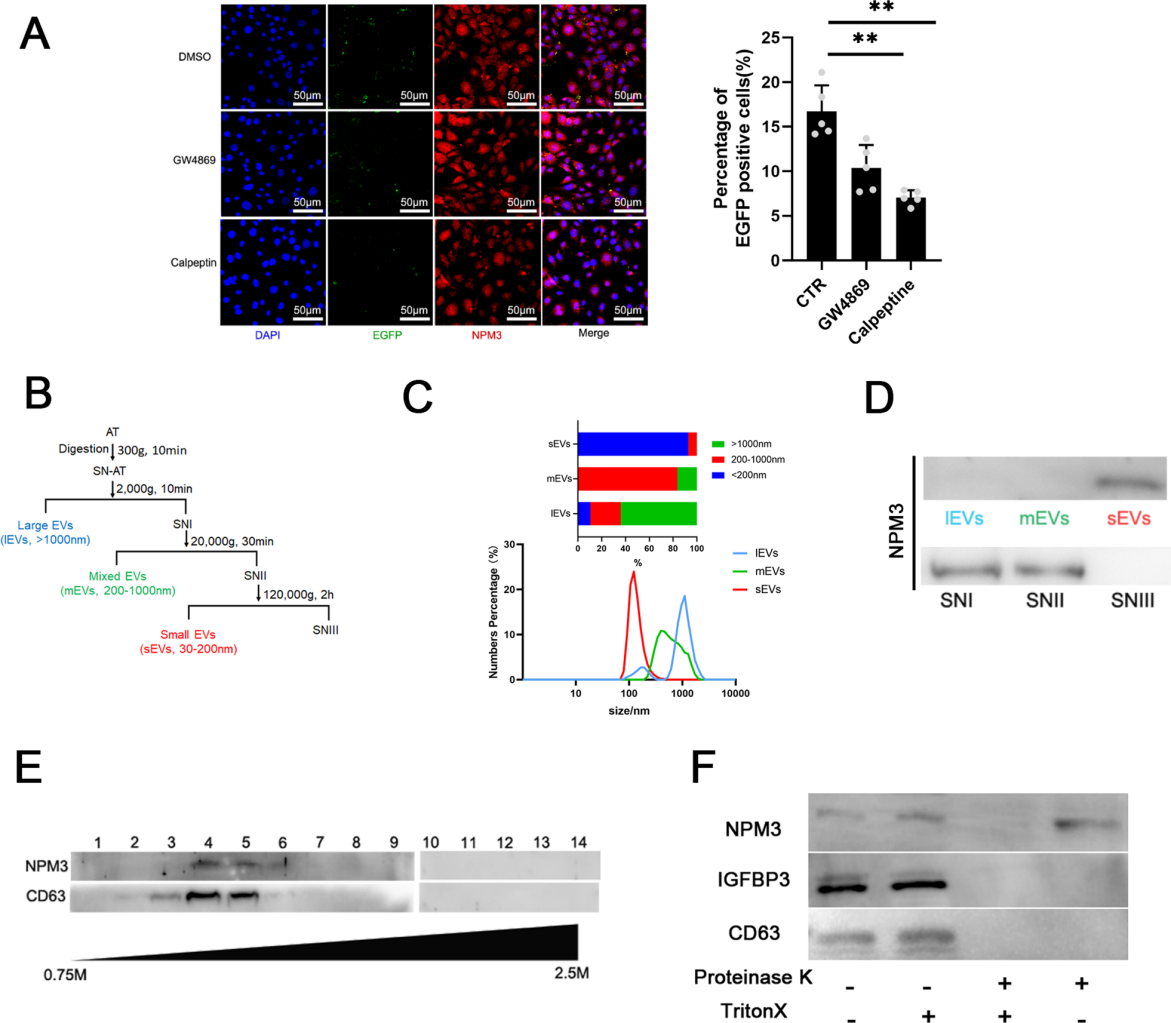
NPM3 was exclusively localized to sEVs. **A** Cell immunofluorescence assay confirming the presence of NPM3-EGFP in recipient cells via the transwell assay, GW4869 or Calpeptin was used to block sEVs release, scale bar = 50 μm; **B** Differential centrifugation procedure for the isolation of sEVs from the supernatants of digested adipose tissue (SN-AT); **C** The size of different types of EVs (lEVs, mEVs, sEVs) were detected using dynamic light scattering; **D** NPM3 expressions in different sizes of EVs and different centrifugations supernatants; **E** Comparison of NPM3 and sEVs marker proteins in 14 fractions (F1–F14) isolated from sucrose density-gradient centrifugation; **F** Comparison of NPM3, CD63, IGFBP3 in sEVs treated with proteinase K and/or Triton-X-100

NPM3 is a protein without a secretory signal peptide, so the detection of NPM3 in plasma indicated that NPM3 was transferred by a certain carrier. In recent years, a transport mechanism of adipokine by sEVs from one tissue to another has drawn much attention as an important mechanism of inter-tissue communications [17, 31]. Our previous study also demonstrated that NPM3 was enriched in sEVs derived from adipose tissue [26]. Thus, we asked whether NPM3 could also be transported by sEVs. To investigate the intercellular transfer of NPM3, NPM3-EGFP fusion protein was expressed in 3T3-L1 preadipocytes, GW4869 or calpeptin was adopted to inhibit the release of sEVs in a transwell assay. The number of EGFP positive cells decreased significantly when inhibitors were added into the transwell culture system (Fig. 3A). To further investigate the localization of NPM3, a differential centrifugation approach was used to isolate different sizes of EVs from adipose tissue extract (ATE) (Fig. 3B). The size distribution of different types of vesicles was determined. The vesicles were divided into different types of vesicles according to the vesicle diameter, the large EVs (> 1000 nm, lEVs), the mixed EVs (200-1000 nm, mEVs) and small EVs (< 200 nm, sEVs) (Fig. 3C). The presence of NPM3 in different samples was determined by western blot, the results indicated that NPM3 was enriched in sEVs not in mixed or large EVs (Fig. 3D). Crude sEVs fraction was also separated on a sucrose gradient cushion to further validate the localization of NPM3, the presence pattern of NPM3 matched to CD63 (sEVs marker) (Fig. 3E). To confirm whether NPM3 was carried by sEVs, sEVs were treated with protease. Upon adding a detergent (Triton-X) to dissolve the lipid bilayer of sEVs, proteinase K treatment was able to eliminate NPM3. In the absence of Triton-X, NPM3 exhibited resistance to proteinase K digestion (Fig. 3F). These findings confirmed that NPM3 was localized to sEVs.

### Knocking down NPM3 in BAT impaired sEVs-BAT mediated WAT browning

**Fig. 4:**
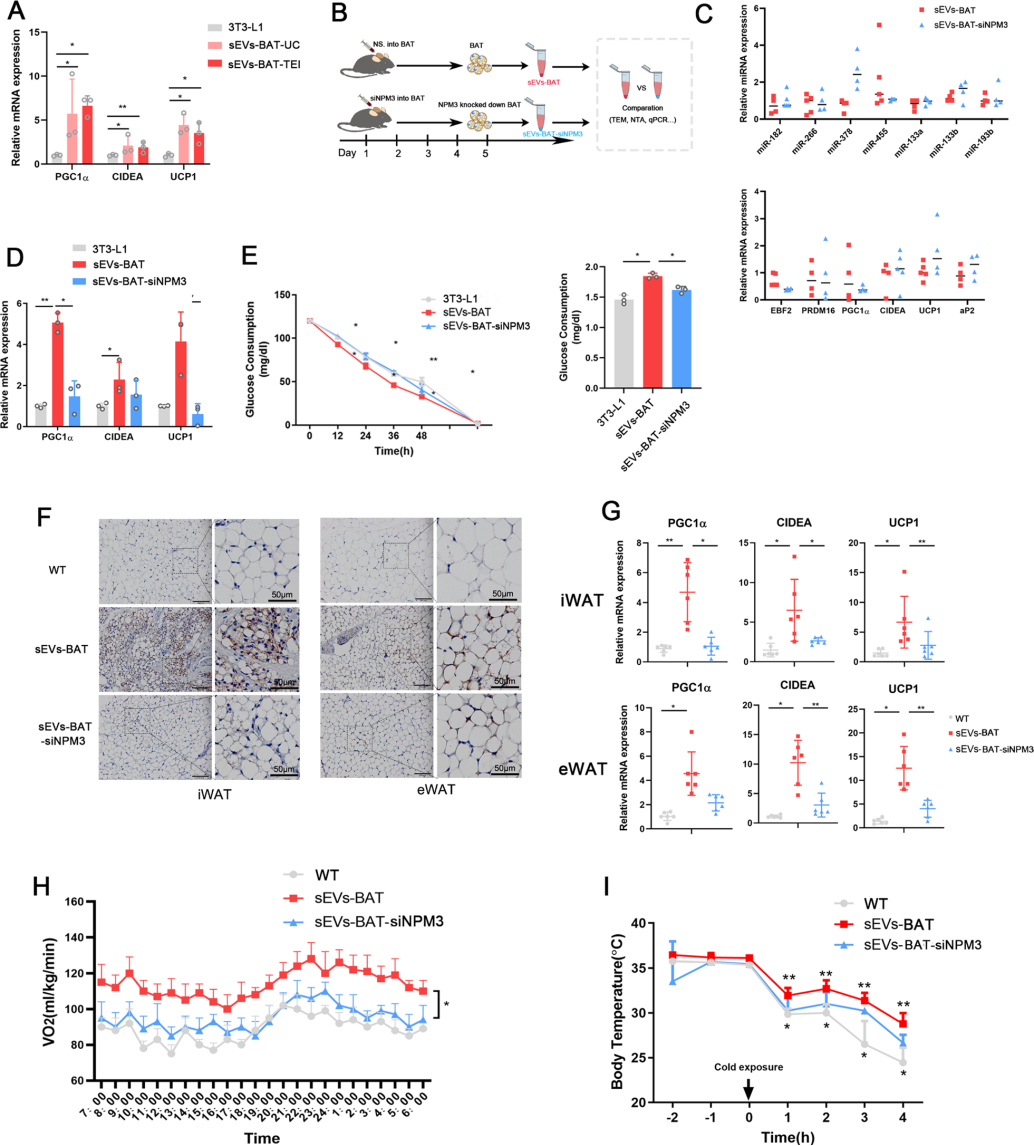
Knocking down NPM3 in BAT impaired sEVs mediated WAT browning. **A** Relative mRNA expressions of PGC-1α, CIDEA and UCP1 in 3T3-L1 preadipocytes treated with sEVs isolated by TEI or ultracentrifugation method (n = 3), data were represented as mean ± SEM and analyzed by student t test, *P < 0.05,, **P < 0.01; **B** A flow chart depicting the isolation of sEVs-BAT and sEVs-BAT-siNPM3; **C** qRT-PCR analysis of thermogenic related mRNA, miRNAs in the sEVs-BAT and sEVs-BAT-siNPM3 (n = 3); **D** Relative mRNA expressions of PGC-1α, CIDEA and UCP1 in 3T3-L1 preadipocytes treated with sEVs-BAT or sEVs-BAT-siNPM3 (n = 3), data were represented as mean ± SEM and analyzed by student t test, *P < 0.05, **P < 0.01; **E** Glucose consumption ratio of 3T3-L1 preadipocytes after treated with sEVs-BAT and sEVs-BAT-siNPM3 (n = 3), data were represented as mean ± SEM and analyzed by student t test, *P < 0.05; **F** Representative UCP1 immunohistochemical staining in iWAT and eWAT in mice treated with sEVs-BAT or sEVs-BAT-siNPM3, Scale bar = 100 μm; **G** Relative mRNA expressions of PGC-1α, CIDEA and UCP1 in iWAT and eWAT in mice treated with sEVs-BAT or sEVs-BAT-siNPM3 (n = 6), data were represented as mean ± SEM and analyzed by one-way ANOVA followed by Tukey’s test, *P < 0.05, **P < 0.01, ***P < 0.001; **H** Determination of oxygen consumption for mice injected with sEVs-BAT or sEVs-BAT-siNPM3 (n = 3), data were represented as mean ± SEM and analyzed by one-way ANOVA followed by Tukey’s, *P < 0.05; **I** Measurement of body temperature for mice injected with sEVs-BAT and sEVs-BAT-siNPM3 under cold treatment over the course of 6 h (n = 4), data were represented as mean ± SEM and analyzed by one-way ANOVA followed by Tukey’s test, *P < 0.05, **P < 0.01, ***P < 0.001

A previous study reported that sEVs-BAT promoted energy expenditure by regulating oxygen consumption in recipient cells [32]. However, it was still elusive whether sEVs-BAT could also regulate WAT browning. sEVs-BAT were isolated by different ultracentrifugation and TEIreagent, co-cultured with 3T3-L1 respectively. sEVs-BAT isolated from two different methods showed an equivalent effect on the promotion of thermogenic genes (Fig. 4A). Considering that NPM3 and sEVs-BAT played a vital role in the regulation of WAT browning, it was reasonable to speculate that downregulation of NPM3 in BAT would affect the browning function of sEVs-BAT. Therefore, we knocked down the expression of NPM3 by siRNA treatment in BAT, then isolated sEVs from treated BAT, named the resulted sample as sEVs-BAT-siNPM3 (Fig. 4B). Knocking down NPM3 in BAT had no obvious impacts on the expressions of thermogenic related miRNAs and mRNAs in sEVs (Fig. 4C) did not alter the quantity and quality of secreted sEVs (Additional file 1: Fig. S7). 3T3-L1 cells and ASCs were treated with sEVs-BAT and sEVs-BAT-siNPM3 respectively, the expressions of the WAT browning marker were significantly impaired in cells treated with sEVs-BAT-siNPM3 (Fig. 4D, Additional file 1: Fig. S8). Consistent with the gene expression results, the increased glucose consumption indicated that the browning of 3T3-L1 cells were also enhancedin sEVs-BAT group whil dewhile decreased with sEVs-BAT-siNPM3 (Fig. 4E). These results confirmed that knocking down of NPM3 in BAT impaired sEVs-BAT mediated white adipocytes browning.

To further investigate the effects of NPM3 on sEV-BAT induced WAT browning in vivo, sEVs-BAT and sEVs-BAT-siNPM3 were injected into the vein of C57BL/6 mice every 3 days separately for 2 weeks. IVIS imaging showed that sEVs were mostly found in the liver of mice (Additional file 1: Fig. S9A), also present in iWAT, eWAT and BAT (Additional file 1: Fig. S9B, C). The expressions of NPM3 in iWAT and eWAT were upregulated when the mice were administrated with sEVs-BAT while there was no obvious change when the mice were administrated with sEVs-BAT-siNPM3 (Additional file 1: Fig. S10). The UCP-1 positive adipocytes in iWAT and eWAT were smaller and multilocular in sEVs-BAT treated mice than that in the sEVs-BAT-siNPM3) group (Fig. 4F). Moreover, the elevated expressions of PGC1α, CIDEA, and UCP1 in the iWAT and eWAT of sEVs-BAT treated mice were impaired by NPM3 knockdown (Fig. 4G). sEVs-BAT injection promoted oxygen consumption of mice while sEVs-BAT-siNPM3 injection did not show a significant difference in oxygen consumption compared to normal mice (Fig. 4H). Moreover, after cold exposure, the mice administrated with sEVs-BAT-siNPM3 had more difficulty maintaining their body temperature than the mice injected with sEVs-BAT (Fig. 4I). These results suggested that knocking down NPM3 in BAT impaired sEVs-BAT mediated WAT browning.

### Knocking down NPM3 in BAT blunted sEVs-BAT mediated obesity combat in HFD-fed mice

**Fig. 5:**
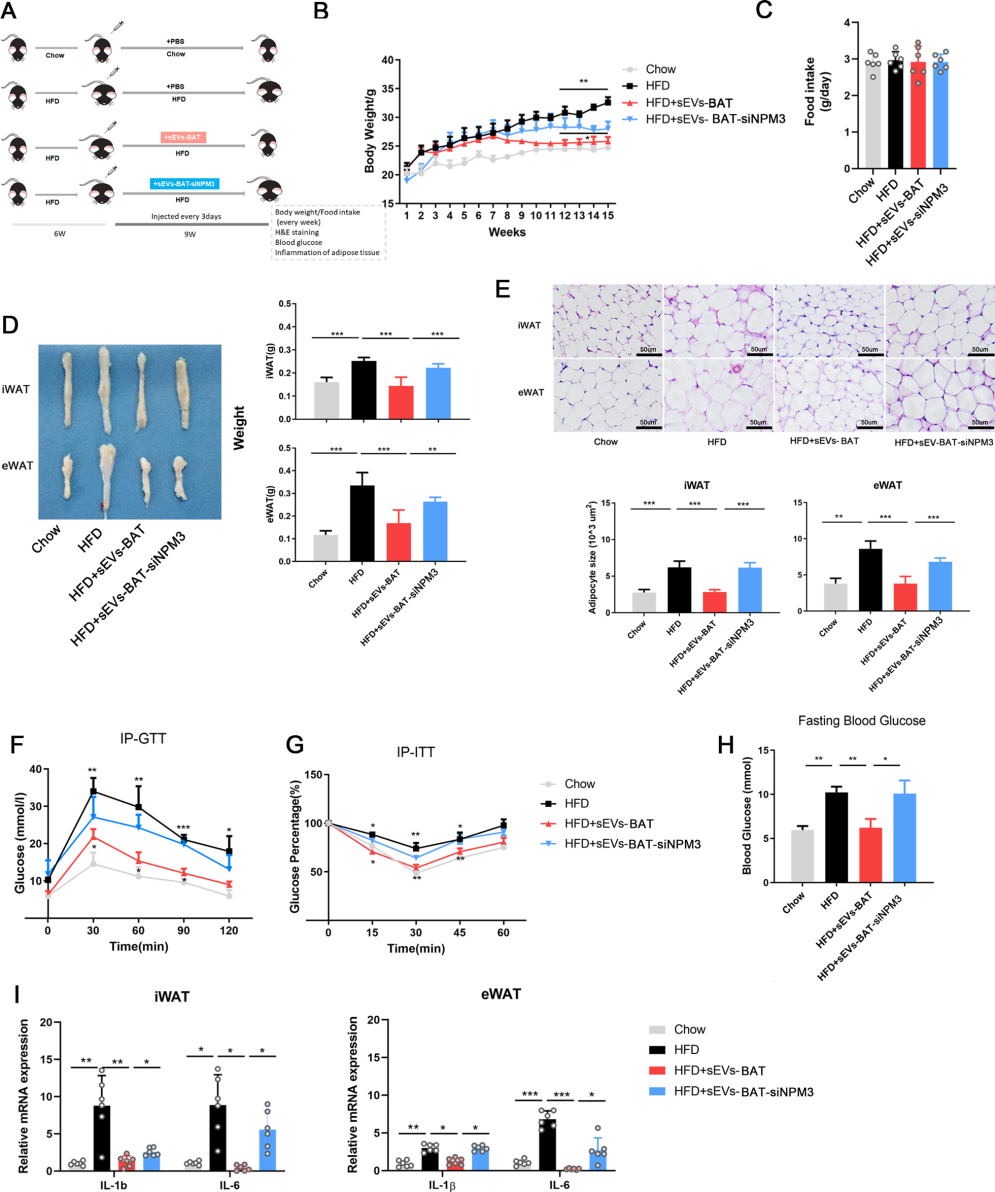
Knocking down NPM3 in BAT blunted sEVs mediated insulin sensitivity in HFD-fed mice. **A** Schematic study plan where 8 weeks old, male C57BL/6 mice were fed with chow or HFD for 15 weeks, HFD-fed mice were injected in vein with sEVs-BAT and sEVs-BAT-siNPM3 during the last 9 weeks; HFD-fed mice treated with PBS were used as controls; **B** Body weight change during the experiment (n = 6) was evaluated after the intervention, data were represented as meanSEM and analyzed by one-way ANOVA followed by Tukey’s test, **P < 0.01; **C** Diet intake (Kcal/group); **D** iWAT, eWAT weight was evaluated after of intervention, data were represented as mean ± SEM and analyzed by one-way ANOVA followed by Tukey’s test, **P < 0.01, ***P < 0.001; **E** H&E staining of iWAT and eWAT were evaluated after of intervention, the size of adipocytes in eWAT of HFD fed mice was evaluated after the intervention (5 images per group), data were represented as mean ± SEM and analyzed by one-way ANOVA followed by Tukey’s test, **P < 0.01, ***P < 0.001; **F** Fasting glucose levels (fasting duration 6 h) were evaluated after the intervention, data were represented as mean ± SEM and analyzed by one-way ANOVA followed by Tukey’s test, *P < 0.05, **P < 0.01, ***P < 0.001; **G** IPGTT and **H** IPITT were performed in mice (n = 6) after the intervention respectively, data were represented as mean ± SEM and analyzed by one-way ANOVA followed by Tukey’s test, P < 0.05, **P < 0.01 versus the HFD group; **I** Relative mRNA expression of IL-6 and IL-1β in iWAT and eWAT of HFD mice were evaluated after the intervention (n = 6), data were represented as mean ± SEM and analyzed by one-way ANOVA followed by Tukey’s test, *P < 0.05, **P < 0.01, ***P < 0.001

A previous study reported that sEVs-BAT could be used to combat obesity, considering the role of NPM3 in WAT browning, we asked whether NPM3 could make a contribution in sEVs-BAT mediated obesity combating. To test this possibility, we injected sEVs-BAT or sEVs-BAT-siNPM3 intravenously into HFD feeding mice for 9 weeks after the mice were induced for obesity successfully (Fig. 5A). sEVs-BAT promoted WAT browning in HFD mice as that in normal fed mice (Additional file 1: Fig. S11). sEVs-BAT injection restricted this HFD-induced weight gain while this effect was attenuated when NPM3 was knocked down in sEVs-BAT (Fig. 5B). Cumulative diet intake was unaltered between the groups (Fig. 5C). sEVs-BAT injected mice significantly attenuated iWAT and eWAT mass as compared to the HFD mice treated with sEVs-BAT-siNPM3 (Fig. 5D). Histological studies indicated that sEVs-BAT administration attenuated HFD-induced adipose hypertrophy while it was not obvious in the sEVs-BAT-siNPM3 treatment group (Fig. 5E). sEVs-BAT administration not only improved glucose tolerance and insulin sensitivity (Fig. 5F, G) but also decreased fasting glucose as well (Fig. 5H). However, these effects were not obvious when the mice were treated with sEVs-BAT-siNPM3. Besides, the significant decrease of pro-inflammatory genes like IL-1β, IL-6 in iWAT and eWAT after sEVs-BAT injection in HFD mice was not obvious in sEVs-BAT-siNPM3 injected HFD mice (Fig. 5I). Conclusively, knocked down NPM3 in BAT impaired sEVs-BAT mediated body weight loss and insulin sensitivity improvement in HFD fed obese mice.

## Discussion

The study of WAT browning has become a hot topic in various acute and chronic metabolic conditions due to that WAT browning might be able to reduce obesity and improve metabolic health [33]. Recent studies have shown that adipokines served as important regulators of BAT development and WAT browning, as illustrated by the classic endocrine hormones, such as FGF21, BMP7, and IGF1 [34, 35]. In this study, we identified a novel adipokine NPM3, which was mainly secreted by BAT, as a crucial component within the thermogenic program to regulate WAT browning both in vitro and in vivo*.* When NPM3 was knocked down in iWAT, the cold-treated mice displayed attenuated thermogenic genes expressions and brown-like adipocytes accumulation. Previous studies also reported that BAT development-related proteins c-Myc and sp1 bind to the promotor of NPM3 to increase its expression [36, 37]. All these results indicated that NPM3 was a positive regulator of WAT browning or BAT development. However, to confirm if NPM3 played a role in the development of brown adipose tissue, further studies are required in adipose tissue-specific NPM3-deficient animal models.

Although the NPM family was found to be associated with a variety of endocrine and metabolic dysregulation, such as aerobic glycolysis, insulin receptor expression, obesity and aging [38–40], the biological functions of NPM3 were largely undefined, especially for the regulation of adipose tissue itself. In this study, we showed that NPM3 altered the expressions of several thermogenic genes in adipose tissues, especially PRDM16. PRDM16, a 140 kDa zinc finger protein, has been demonstrated to play a major role in brown/beige adipocyte development [41] and binds to many regulatory factors (e.g., PGC-1α, PGC-1β, C/EBPβ, CtBPs) to stimulate brown adipogenesis [41]. In this study, we demonstrated that NPM3 upregulated PRDM16 expression by stabilizing PRDM16 mRNA. Since RNA binding protein regulates distinct steps of mRNA biogenesis including 5′ capping, pre-mRNA splicing, 3′mRNA cleavage and polyadenylation, mRNA export, mRNA editing and methylation, mRNA decay and translation, and mRNA localization [42], NPM3 may affect multiple RNA processing steps, including but not limited to RNA stability. The precise mechanism of NPM3 should be studied in the future.

Brown fat contributes to whole-body energy homeostasis through not the thermogenesis-dependent role of brown fat but its release of secreted factors [43]. We revealed that NPM3 was exclusively included in sEVs-BAT. Considering that NPM3 was a protein that without a signal peptide, sEVs might be a vital way for NPM3 to take part in intercellular communication. Knocking down of NPM3 in BAT was not only decreased the expression of NPM3 in sEVs-BAT but also impaired sEVs-BAT mediated WAT browning. This indicated that NPM3 might partly play a vital role in maintaining the functions of sEVs-BAT. Further research was needed to illustrate the role of NPM3 in sEVs-BAT through constructing NPM3 completely depleted sEVs.

Moreover, although we found that sEVs-BAT could promote WAT browning, the effects of sEVs-BAT on the other metabolic organs were still elusive. In our study, we noted that sEVs-BAT were not only internalized by adipose tissue but also by the liver. It is well known that the development of diabetes entails alterations in insulin-sensitive tissues such as the liver, the skeletal muscle, and the adipose depots, leading to a state of glucose intolerance [44]. Therefore, further study is needed to demonstrate whether sEVs-BAT exert metabolic benefits on the other peripheral tissues. Many studies have attempted to increase WAT browning and BAT activity through the use of several activators of thermogenesis for the prevention and treatment of obesity and obesity-related metabolic syndrome [45]. At present, there are several methods to enhance the total mass and/or activity of BAT including BAT/brown adipocytes transplantation, BAT tissue engineering, WAT browning induction through medicine. While β3-adrenergic receptor agonists have been proved to facilitate the browning of WAT in mice, challenges were still existed due to undesirable side effects on the cardiovascular system when used in humans [46]. Moreover, we found that injection of sEVs-BAT could also promote WAT browning in vivo and has a pivotal effect in combating HFD-induced obesity and whole-body glucose homeostasis by increasing energy expenditure and reducing adipose inflammation. Considering that sEVs are completely cell-free and hypoimmunogenic, sEVs could be an important tool for BAT engineering and engineered sEVs would be more suitable for clinically translatable therapy compare to obtaining sEVs directly from BAT. For example, the cells (e.g., ASCs) could be engineered to overexpress NPM3 using either non-viral or viral methods and then isolated the NPM3 carried sEVs for treatment [47–49].

## Materials and methods

### Animals

All animal experiments were performed according to procedures approved by the Ethical Committees of the State Key Laboratory of Oral Diseases, West China School of Stomatology, Sichuan University (approved in 2017, approval number WCHSIRB-D-2017-183). 8-week-old male C57BL/6 mice were purchased from Chengdu DaShuo Biotechnology Co., Ltd. 10-week-old male wild type (WT) C57BL/6 mice and 10-week-old male obese C57BL/6 (ob/ob) mice were purchased from Model Animal Research Center of Nanjing University. Animals were housed in a plexiglass cage (5 per cage) at a temperature (22 ± 3 °C) and humidity (55 ± 15%). Animals were provided with food and sterile water and kept on a 12-h light–dark cycle acclimated for 1 week before the study. β3-adrenergic agonist CL-316,243 (Sigma, U.S.A.) was intraperitoneally injected into mice every day at 1 mg/kg bodyweight for 5 days. For acute cold exposure, mice were individually caged with food withdrawn and water provided, placed in a 4 °C cold room, and core body temperature was measured with a thermometer (Taishi, TES-1310, China). The experiment was conducted in a random manner. All the mice of the same sex and weight in a certain range were randomly divided into three groups. Four animals of the same sex in the same nest and with similar body weight were used as the compatibility group. After the allocation, the number of animals in each group was equal. The weight of each group was similar, so as to reduce the experimental error. According to the allocation of different stages of the experiment, there are corresponding records on the label outside the cage. Correspondingly, the result evaluation and data analysis are analyzed according to the random cage unit.

### Preparation of plasma

Plasma was collected from the tail vein (mice or rat) with a syringe pre-treated with heparin sulfate. Blood was span down at 2000*g*, 20 min at room temperature. 500 μl of freshly collected plasma was incubated with 500 μl of 2× sample buffer at 95℃ for 10 min. Before the analysis, 1 μl of each sample was added to 49 μl of 1× sample buffer and further incubated at 95 °C for 10 min and then were used to perform SDS-PAGE.

### In vivo adenovirus associated virus injection

Adenovirus associated virus (AAV) expressing N-terminally EGFP-tagged NPM3 genes (AAV-NPM3-EGFP) was designed and synthesized by Hanbio Co. Ltd. AAV containing only EGFP (AAV-CTR) was used as a negative control. For in vivo injection, 8-week-old male wild-type C57BL/6 mice were used. The interscapular brown adipose tissue (BAT) was injected with AAV at 5 different sites in each side with 1.0 × 10^^10^ transducing units per site to cover the whole tissue. Two weeks after injection, the mice were sacrificed and the BAT tissues were obtained. BAT transplantation was carried out as previously described1. In brief, AAV-NPM3-EGFP BAT was isolated and cut into several pieces and transplanted into 8-week-old male C57BL/6 mice. For each recipient mouse, a total of 1.0 g of the resulting slices of fat were transplanted into the inguinal area. One week later, the mice were sacrificed and the iWAT, adjacent muscle, liver, lung, kidney were obtained for histology analysis.

### 3T3-L1 preadipocytes differentiation and treatment

3T3-L1 preadipocytes were obtained from Kunming Cell Bank, Chinese Academy of Sciences and maintained in DMEM with 10%FBS. For adipogenic induction, 3T3-L1 preadipocytes were incubated with 10% FBS-DMEM medium supplemented with 0.5 mM isobutylmethylxanthine (Sigma, USA), 1 mM dexamethasone (Sigma, USA) and 5 mg/ml insulin (Merck, USA) for five days [50]. Then the cells were treated with induction medium supplemented with rosiglitazone (2 μM) for browning induction for another five days.

#### Lentivirus infection

Lentivirus expressing the entire coding sequence of murine NPM3 was designed and synthesized by GeneCopoeia Co. Ltd. Lentivirus containing only EGFP (empty vector) was used as a negative control. 3T3-L1 adipocytes (5 × 10^4^ per well) were seeded in 24-well plates and cultured overnight. The cells were infected with lentivirus (MOI = 100) using polybrene at a concentration of 5 μg/ml. After infection, the cells were selected with puromycin (1 μg/ml) to generate stable NPN3-expressing preadipocytes.

### siRNA transfection

3T3-L1 preadipocytes (5 × 10^4^ per well) were seeded in 24-well plates and cultured overnight. The cells were transfected with 50 nM non-targeting siRNA or three different NPM3-targeting siRNAs (Ruibo, China) using the Lipofectamine 3000 transfection reagent following the manufacturer’s instructions (Life Technologies, USA). The negative control group was treated only with the transfection reagent. After transfection, cells were used for RNA extraction for qPCR. The knockdown efficiency of NPM3 targeting siRNAs was evaluated by qPCR 72 h post-transfection. siRNA #2 and siRNA #3 showed > 90% inhibition of NPM3 mRNA expression compared to the control siRNA. Therefore, NPM3 siRNA #2 and #3 was used in all of the experiments. In vivo study, 15 nmol Cholesterol-modified siNPM3 (Ribobio) dissolved in diluted water were injected directly into the BAT of 8 week-old male C57BL/6 mice (n = 3) every day by local injection at multiple points. 5 days later, BAT was collected for sEVs isolation.

### Glucose consumption measurements

The cell culture medium of 3T3-L1 preadipocytes after browning induction was collected at different time points (0, 12, 24, 36, 48 h). The concentration of glucose in the medium was determined using EnzyChrom™ Glucose Assay Kit (BioAssay, USA) following the manufacturer’s instructions. The absorbance was measured at 570 nm with a spectrophotometer (MultiskanGO, Thermo Scientific).

### RNA immunoprecipitation (RIP) assay

3T3-L1 preadipocytes (5 × 10^4^ per well) were seeded in 24-well plates and cultured overnight. FLAG-NPM3 fusion protein expression plasmids and empty plasmids with the same backbone (GeneCopoeia, USA) were used. The cells were transfected with 1 μg plasmids using the Lipofectamine 3000 transfection reagent (Life Technologies, USA) following the manufacturer's instructions. After 48 h, the cells were used to perform a RIP experiment using an anti-FLAG antibody (CST, USA) or isotype-matched control antibody (normal rabbit IgG; Sigma). Following the recovery of antibodies using protein A/G beads, qRT-PCR was performed on the precipitates to detect the gene expression. RNA immunoprecipitation (RIP) assay was performed using a Magna RIP Kit (EMD Millipore, Billerica, MA, USA) according to the manufacturer’s instructions.

### mRNA stability analysis

NPM3 overexpressed 3T3-L1 preadipocytes cells (5 × 10^4^ per well) were induced for browning in a 24-well plate for 10 days. 3T3-L1 preadipocytes cells (5 × 10^4^ per well) were also treated with siNPM3 for 2 days and subsequently induced for browning in a 24-well plate for 10 days. Then cells were treated with actinomycin D (5 mg/ml), total intracellular RNA was harvested at different times (0, 1, 2, 3, 4 and 5 h). qRT-PCR analysis was performed to calculate relative mRNA expression using the 2^−ΔΔCT^ method. mRNA levels were calibrated to the 0 h time point.

### sEVs isolation

sEVs used in this study were isolated using the Total Exosome Isolation (TEI) reagent with minor modification. Briefly, 5 g of adipose tissue were collected from 8-week-old male C57BL/6 mice, washed extensively with sterile phosphate-buffered saline (PBS) to remove the debris and red blood cells. The tissue was cut into small pieces (1–2 mm^3^) under aseptic condition and then treated with 10 ml 0.075% collagenase (type I) for 30 min at 37 °C. The digested adipose tissue was centrifuged at 300*g* for 10 min and the supernatant (SN-AT) was collected, filtered (0.22 μm filter) to remove the debris of cells. Then the supernatant was concentrated with Amicon^®^ Ultra-15 Centrifugal Filter Units (10,000 Mw cut off the membrane, Millipore, USA) at the speed of 5000*g* for 30 min (4 °C, Beckman Avanti J-26S XP centrifuge, JS5.30). The concentrated medium was mixed with 0.5 volume of Total Exosome Isolation™ reagent (Life Technologies, USA), incubated overnight at 4 °C and spun down for 1 h at 10,000 g at 4 °C. The pellet was re-suspended in 100 μL and used for cell treatment or injection in vivo.

### EV analysis with sequential centrifugation

The supernatant from digested adipose tissue (SN-AT) was analyzed by sequentially centrifuged. SN-AT was firstly centrifuged at 2000*g* for 10 min to collect large EVs (lEVs), then the supernatant (SNI) was collected and further centrifuged at 20,000*g* for 30 min [4 °C, Beckman Avanti J-26S XP centrifuge, JA25.50, polyamide tube (Cat. 357,003)]. The pellet was collected as mixed EVs (mEVs), and the supernatant (SNII) was further ultracentrifuged at 120,000*g* for 2 h [4 °C, Himac CP 70MX centrifuge, P40ST, Kadj:328.96, polyamide tube (Cat. 332901A)] to collect sEVs. The supernatant (SNIII) was also collected for western blot analysis. The pellets in every centrifugation step were collected and re-suspended in 100μL PBS for further analysis.

### Transwell assays

Recipient cells were seeded into 6-well tissue culture plates (Corning, NY, USA) at a density of 2.5 × 10^4^ cells per well and allowed to attach overnight. Costar 24 mm Transwell® Permeable Support Inserts with 0.4 µm Polyester Membranes (Corning) were placed on top of each well. Donor cells were seeded into the inserts at a density of 2 × 10^5^ cells per insert and made up to 1.5 ml final volume of culture medium. For GW4869 (20 μM, Selleck, USA) or calpeptine (50 nM, MCE, USA) treatment, the compounds were diluted appropriately in DMSO and added to the culture media in the inserts. The plates were incubated for 48 h at 37 °C, 5% (v/v) CO_2_.

### Immunofluorescence

2.5 × 10^4^ 3T3-L1 cells were seeded into Confocal Dish (Martinsried, Germany) and allowed to attach overnight. Cells were fixed with 4% (w/v) paraformaldehyde and permeabilized with 0.3% (v/v) Triton X-100 for 5 min, blocked with 5% (w/v) bovine serum albumin in PBS for 1 h at room temperature and incubated with primary antibodies overnight at 4 °C followed by secondary antibodies for 2 h. The primary antibodies and dilutions are NPM3 (1:200). The secondary antibodies and dilutions are Alexa-555 conjugated anti-rabbit (1:300). Cells were imaged on an Olympus FV1000 confocal microscope. Images were stacked to ensure equal adjustments to all images.

### Proteinase K digestion assay

sEVs (TEI reagent precipitation) were collected and exposed in suspension to either 1 μg/μl Proteinase K (Sigma), or 0.3%v/v Triton-X100, or both, or neither for 15 min on ice. After exposure, all samples were incubated with 1 mM PMSF proteinase inhibitor (KeyGEN, China) for 15 min, before undergoing normal Western blot.

### Induction of 3T3-L1 preadipocytes using sEVs

3T3-L1 preadipocytes were plated in 24-well plates at a density of 10^5^cells/well, cultured for 24 h, then rinsed with PBS and incubated with 2 ml of one of three different culture medium for up to 10 days. The medium were: (1) basal medium [DMEM supplemented with 10% fetal bovine serum (FBS)], as a negative control; (2) basal medium supplemented with sEVs-BAT isolated by TEI method (50 μg/ml); (3) basal medium supplemented with sEVs-BAT-siNPM3 isolated by TEI method (50 μg/ml). The medium was changed every 3 days. The cells were collected on day 10 for qRT-PCR analysis.

### sEVs injection in vivo

Eight-week-old male C57BL/6 mice (purchased from Chengdu DaShuo Biotechnology Co., Ltd) were divided into three groups (n = 4). They were injected via the tail vein with sEVs-BAT or sEVs-BAT-siNPM3 (TEI method) (2 μg sEVs/g body weight, resuspended in 200 μl PBS) from the digested BAT every 2 days and lasted for 2 weeks. The control group (WT) was injected with 200 μl PBS. 2 weeks later, O_2_ consumption was detected and the iWAT and eWAT were collected for Immunochemical (IHC) staining and qRT-PCR analysis.

To evaluate the effects of sEVs on obesity, a normal diet containing 10% kcal fat (RDI, D12450J) and a high-fat diet containing 60% kcal (RDI, D12492) were purchased from Research Diets, Inc. 8 week-old male C57BL/6 were fed with a high-fat diet for 6 weeks firstly, at 7th week, the weight was approximately 20% more than the normal diet-fed mice, they were injected via the tail vein with sEVs-BAT or sEVs-BAT-siNPM3 (TEI method) (2 μg sEVs/g body weight, resuspended in 200 μl PBS, n = 6) isolated from the digested BAT every 2 days and lasted for 9 weeks. Bodyweight, food intake was monitored weekly. After 9 weeks of injection, glucose tolerance, insulin sensitivity was determined and iWAT and eWAT were collected for immunochemical (IHC) staining and qRT-PCR analysis.

### Oxymax metabolic analysis

sEVs treated mice were acclimated for 12 h in the metabolic cages, and their metabolic rates were measured for 24 h in an indirect open-circuit calorimeter (Oxymax Comprehensive Lab Animal Monitoring System; Columbus Instruments). O_2_ consumption was measured at room temperature (RT) and normalized to body weight to account for the disparity in body weight between the groups.

### Glucose tolerance test (GTT) and insulin tolerance test (ITT)

For GTT, mice were fasted for 8 h. After basal glucose measurement, glucose (2 g/kg, i.p.) was injected and blood glucose was measured from the tail tip at 15, 30, 60, 90 and 120 min by using a glucometer (Accu-Chek, Roche Diagnostics). For ITT, mice were fasted for 5 h. Following basal glucose measurement at 0 min time point, insulin (0.75 U/kg, i.p.) was injected in both groups and blood glucose from the tail tip was measured at 15, 30, 60, 90, and 120 min.

### Immunochemical (IHC) staining

iWAT, eWAT, BAT, liver, and muscle tissues were fixed in 10% neutral-buffered formalin for 24 h. Tissues were embedded in paraffin and sectioned at 4 μm. For IHC, tissues were incubated for 2 h at 60 °C, deparaffinized, and rehydrated. Antigen retrieval was performed using citrate buffer (pH6) at 97 °C for 20 min. Endogenous peroxidase activity was blocked by incubating the sections with 3% hydrogen peroxide for 10 min at room temperature. Non-specific binding of the antibody was blocked by incubating the slides with 5% normal goat serum in PBS containing 0.1% Tween 20 (PBST) for 1 h at room temperature. The slides were then incubated with primary antibodies against NPM3 (1:200, Zen Bioscience, China), UCP1 (1:200, Abcam, U.K.) overnight at 4 °C. After washing, each slide was incubated with the appropriate HRP-labeled secondary antibody, and signals were developed with DAB solution before counterstaining with hematoxylin.

### Western blot analysis

Total proteins were extracted by the Total Protein Extraction Kit (KeyGEN, China). 30 μg proteins were dissolved in RIPA Lysis Buffer (KeyGEN, China), resolved on a 10% polyacrylamide gel and blotted onto PVDF membrane. The membranes were blocked for 1 h and then incubated with primary antibodies (listed in Additional file 2: Table S1) at 4 °C overnight, followed by horseradish peroxidase (HRP)-conjugated secondary antibodies for 1 h at room temperature. Immobilon Western Chemiluminescent HRP Substrate (Millipore, USA) was used for the detection following the manufacturer’s instructions. Signals were visualized by ImageQuant LAS4000 mini (GE Healthcare, USA). Band intensities were determined using Image J software and normalized to internal control ACTB.

### qRT-PCR

Total cellular RNA was extracted using RNAiso Plus (TaKaRa Biotechnology) according to the manufacturer’s instructions. The quantity of RNA was calculated based on the absorbance at 260 nm detected by a NanoDrop 2000 spectrophotometer. 260/280 nm absorbance ratio between 1.8 and 2.0 was considered as good purity RNA and used for further experiment. cDNA was reverse transcribed from 2 µg of RNA with First Strand cDNA Synthesis Kit (Thermo Scientific, USA) according to the manufacturer’s instructions with a final volume of 50 µl. 1 µl out of 50 µl reverse transcribed cDNA was used as a template for qPCR with iTaq™ Universal SYBR^®^ Green (BioRad, USA) utilizing Eco Real-time PCR System (Illumina, USA). Reaction conditions were: 95 °C for 2 min; followed by 40 cycles of 95 °C for 5 s, 60 °C for 30 s. The results were analyzed using the 2 − ΔΔCT relative quantitative method with ACTB as an internal control. Primer sequences are listed in Additional file 2: Table S2.

### Statistical analysis

Results are presented as mean ± SEM. All statistical tests were performed using GraphPad Prism5. Significance between the two groups was assessed by Student’s t-test. The comparisons between multiple groups were carried out using one-way ANOVA followed by Tukey’s test. Linear regression analysis was used to analyze plasma NPM3 levels of mice across different body weight groups. Sample sizes and other statistical parameters are indicated in the figures and texts. *p < 0.05, **p < 0.01, ***p < 0.001. Significance was concluded at p < 0.05.

## Supplementary Information

**Additional file 1: Figure S1. **Expressions of NPM3 in the plasma (left) and iWAT (right) when mice were under cold exposure.** Figure S2. **Effects of NPM3 on brown preadipocytes differentiation Relative NPM3 mRNA levels after 3T3-L1 preadipocytes were infected with NPM3 overexpression lentiviruses/empty vector (CTR) (A) or transfected with NPM3 siRNA/siRNA-Control (CTR) (B) (n=3), data were represented as mean±SEM and analyzed by student t test, *P<0.05, **P<0.01, ***P<0.001. **Figure S3. **Browning induction of 3T3-L1 cells. (A) Expressions of browning-related markers (n=3), data were represented as mean±SEM and analyzed by student t test, *P<0.05, **P<0.01, ***P<0.001.; (B) Oil red O staining.** Figure S4. **Effects of NPM3 on the browning of ASCs from WAT and BAT. Relative NPM3 mRNA levels after brown ASCs (bASCs) were infected with NPM3 overexpression lentiviruses (A) or transfected with NPM3 siRNA (B) and differentiated into adipocytes; (C) Relative mRNA expressions of PGC-1α, CIDEA and UCP1 at the late stage of bASCs differentiation (n=3); Relative NPM3 mRNA levels after white ASCs (wASCs) were infected with NPM3 overexpression lentiviruses (D) or transfected with NPM3 siRNA (E) and differentiated into adipocytes (n=3); (F) Relative mRNA expressions of PGC-1α, CIDEA and UCP1 at the late stage of wASC differentiation. (n=3). Data represent mean±SEM, *P<0.05, Student t test. **Figure S5. **Effects of NPM3 on cold-induced WAT browning. (A) A flow chart depicting the in vivo knocking down NPM3 and browning induction in iWAT to confirm the functions of NPM3 in cold induced WAT browning; (B) Influences of NPM3 on the expressions of browning-related genes in iWAT after cold exposure (n=3), data were represented as mean±SEM and analyzed by student t test, *P<0.05, **P<0.01; (C) IHC staining of UCP1 in iWAT. **Figure S6. **The subcellular distribution of NPM3 in 3T3-L1cells during browning induction. 3T3-L1 preadipocytes were induced for adipocytes for five days. Then the cells were treated with an induction medium supplemented with rosiglitazone for browning induction for another five days. (A)The subcellular distribution of NPM3 was detected by immunofluorescence (IF) on day 0,3,5,10 (scale bar=50μm); (B) Western blot was used to detect the expressions of NPM3 in the cytoplasm and nuclear of 3T3-L1 cells on day 0,3,5,10. **Figure S7. **Characterization of sEVs-BAT and sEVs-BAT-siNPM3. (A) WB and semi-quantification analysis of NPM3 expression in sEVs-BAT and sEVs-BAT-siNPM3 (n=3), data were represented as mean±SEM and analyzed by student t test, **P<0.01; (B) sEVs-BAT and sEVs-BAT-siNPM3 release in BAT; (C) Electron micrograph of sEVs-BAT and sEVs-BAT-siNPM3, scale bar=200nm; (D) The size distribution of sEVs-BAT and sEVs-BAT-siNPM3 as determined by NTA; (E) 3T3-L1 cells were incubated with DiO-labeled sEVs-BAT and sEVs-BAT-siNPM3 (green) and stained with phallotoxins (red), nuclei were stained with DAPI (blue), scale bar=50μm. **Figure S8. **Effects of sEVs-BAT and sEVs-BAT-siNPM3 on the browning induction of ASCs derived from white adipose tissue (wASC). sEVs-BAT and sEVs-BAT-siNPM3 (50μg/ml) were co-cultured with wASCs for 10 days and relative mRNA expressions of PGC-1α, CIDEA and UCP1were detected by qRT-PCR, (n=3), data were represented as mean±SEM and analyzed by student t test, *P<0.05, **P<0.01.** Figure S9. **Distribution of sEVs-BAT and sEVs-BAT-siNPM3 in vivo. (A) Representative images of sEVs-BAT and sEVs-BAT-siNPM3 distribution in mice harvested at 6 hours following i.v. infusion of DiR labeled sEVs; (B) Representative images of different organs distribution (lung, heart, liver, spleen, muscle, kidney) 6 hours post-injection of DiR labeled sEVs-BAT and sEVs-BAT-siNPM3; (C) Representative images of adipose tissue (iWAT, eWAT, BAT) 6 hours post-injection of DiR labeled sEVs-BAT and sEVs-BAT-siNPM3. **Figure S10. **Effects of sEVs-BAT or sEVs-BAT-siNPM3 on the expressions of NPM3 in iWAT and eWAT. Mice were injected via the tail vein with sEVs-BAT or sEVs-BAT-siNPM3 (2μg sEVs/g body weight) every 2 days and lasted for 2 weeks. The expressions of NPM3 in iWAT (left) and eWAT (right) were detected by western blot (n=3), data were represented as mean±SEM and analyzed by one-way ANOVA followed by Tukey’s test, *P<0.05, **P<0.01, ***P<0.001. **Figure S11. **Knocking down of NPM3 impaired sEVs-BAT mediated WAT browning in the HFD mice. The mice were fed with a high-fat diet for 6 weeks firstly, then they were injected via the tail vein with sEVs-BAT or sEVs-BAT-siNPM3 (2μg sEVs/g body weight, n=6) isolated from the digested BAT every 2 days and lasted for 9 weeks. The expressions of browning related genes in iWAT and eWAT were detected by qRT-PCR (n=6), data were represented as mean±SEM and analyzed by one-way ANOVA followed by Tukey’s test, *P<0.05, **P<0.01, ***P<0.001.

**Additional file 2:** **Table S1. **Antibodies.** Table S2.** Primers for qRT-PCR.

## Data Availability

The data sets generated during the current study are available from the corresponding author on reasonable request.

## Abbreviations

NPM3: Nucleoplasmin3
sEVs: Small extracellular vesicles
sEVs-AT: Small extracellular vesicles derived from adipose tissue
ASCs: Adipose derived stromal/stem cells
WAT: White adipose tissue
BAT: Brown adipose tissue
PGC-1α: Peroxisomeproliferator-activated receptor γ-coactivator 1α
CIDEA: Cell death-inducing DNA fragmentation factor alpha-like effector A
UCP1: Uncoupling protein 1
RBP: RNA binding protein
RIP: RNA immunoprecipitation
PRDM16: PR domain-containing protein 16
HFD: High fat diet
EVs: Extracellular vesicles
iWAT: Inguinal white adipose tissue
eWAT: Epididymal white adipose tissue
