# Supplementary material for: Nucleophosmin3 carried by small extracellular vesicles contribute to white adipose tissue browning
Source: J Nanobiotechnology. 2022 Mar 28;20:165. doi: 10.1186/s12951-022-01381-1 (PMC8961928; doi:10.1186/s12951-022-01381-1)
Supplement: Supplementary file 1 — Additional file 1: Figure S1. Expressions of NPM3 in the plasma (left) and iWAT (right) when mice were under cold exposure. Figure S2. Effects of NPM3 on brown preadipocytes differentiation Relative NPM3 mRNA levels after 3T3-L1 preadipocytes were infected with NPM3 overexpression lentiviruses/empty vector (CTR) (A) or transfected with NPM3 siRNA/siRNA-Control (CTR) (B) (n=3), data were represented as mean±SEM and analyzed by student t test, *P<0.05, **P<0.01, ***P<0.001. Figure S3. Browning induction of 3T3-L1 cells. (A) Expressions of browning-related markers (n=3), data were represented as mean±SEM and analyzed by student t test, *P<0.05, **P<0.01, ***P<0.001.; (B) Oil red O staining. Figure S4. Effects of NPM3 on the browning of ASCs from WAT and BAT. Relative NPM3 mRNA levels after brown ASCs (bASCs) were infected with NPM3 overexpression lentiviruses (A) or transfected with NPM3 siRNA (B) and differentiated into adipocytes; (C) Relative mRNA expressions of PGC-1α, CIDEA and UCP1 at the late stage of bASCs differentiation (n=3); Relative NPM3 mRNA levels after white ASCs (wASCs) were infected with NPM3 overexpression lentiviruses (D) or transfected with NPM3 siRNA (E) and differentiated into adipocytes (n=3); (F) Relative mRNA expressions of PGC-1α, CIDEA and UCP1 at the late stage of wASC differentiation. (n=3). Data represent mean±SEM, *P<0.05, Student t test. Figure S5. Effects of NPM3 on cold-induced WAT browning. (A) A flow chart depicting the in vivo knocking down NPM3 and browning induction in iWAT to confirm the functions of NPM3 in cold induced WAT browning; (B) Influences of NPM3 on the expressions of browning-related genes in iWAT after cold exposure (n=3), data were represented as mean±SEM and analyzed by student t test, *P<0.05, **P<0.01; (C) IHC staining of UCP1 in iWAT. Figure S6. The subcellular distribution of NPM3 in 3T3-L1cells during browning induction. 3T3-L1 preadipocytes were induced for adipocytes for five days. Then the cells were treat [file 12951_2022_1381_MOESM1_ESM.docx]

**Additional file 1**

Nucleophosmin3 Carried by Small Extracellular Vesicles Contribute to White Adipose Tissue Browning

Yan Zhang^2,3,5,#^, Mei Yu^1,2,#,*^, Jia Dong^1,2,3^, Yue Wu^1,2,3^, Weidong Tian^1,2,3,*^

1.State Key Laboratory of Oral Disease & National Clinical Research Center for Oral Diseases, West China School of Stomatology, Sichuan University, Chengdu, China

2.National Engineering Laboratory for Oral Regenerative Medicine, West China School of Stomatology, Sichuan University, Chengdu, China

3. Engineering Research Center of Oral Translational Medicine, Ministry of Education, Sichuan University, Chengdu, China

4. Department of Oral and Maxillofacial Surgery, West China Hospital of Stomatology, Sichuan University, Chengdu, China.

5. Department of Oral and Maxillofacial Surgery, Tianjin Stomatological Hospital, School of Medicine, Nankai University, Tianjin, China

#These authors contributed equally.

Corresponding Author

*Correspondence: yumei925@hotmail.com; Tel.: +86-28-85503499；drtwd@sina.com; Tel.: +86-28-85503499；

**Additional Figures**

**
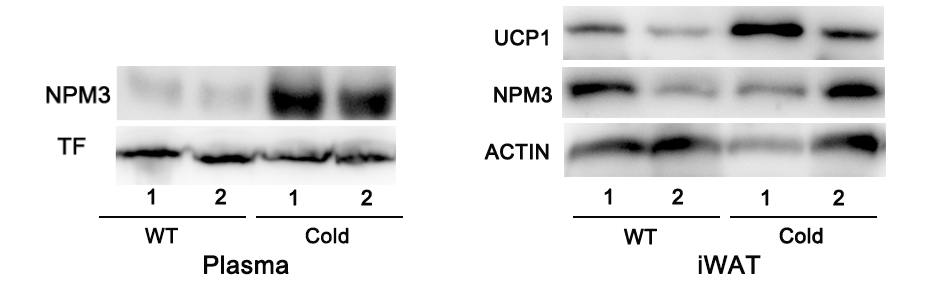
**

**Figure S1. Expressions of NPM3 in the plasma (left) and iWAT (right) when mice were under cold exposure.**


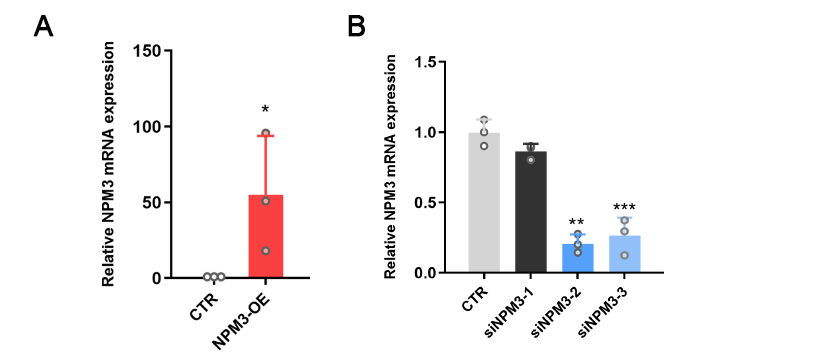


**Figure S2 Effects of NPM3 on brown preadipocytes differentiation** Relative NPM3 mRNA levels after 3T3-L1 preadipocytes were infected with NPM3 overexpression lentiviruses/empty vector (CTR) (A) or transfected with NPM3 siRNA/siRNA-Control (CTR) (B) (n=3), data were represented as mean±SEM and analyzed by student t test, *P<0.05, **P<0.01, ***P<0.001.

**
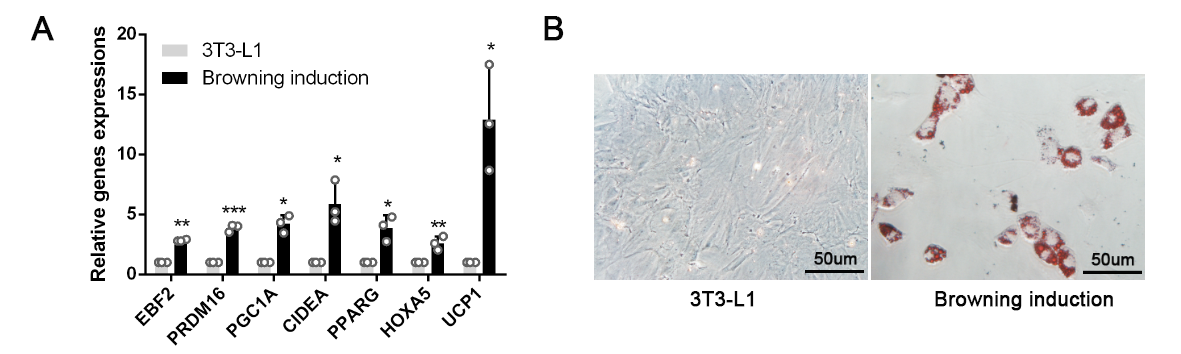
**

**Figure S3. Browning induction of 3T3-L1 cells.** (A) Expressions of browning-related markers (n=3), data were represented as mean±SEM and analyzed by student t test, *P<0.05, **P<0.01, ***P<0.001.; (B)Oil red O staining.


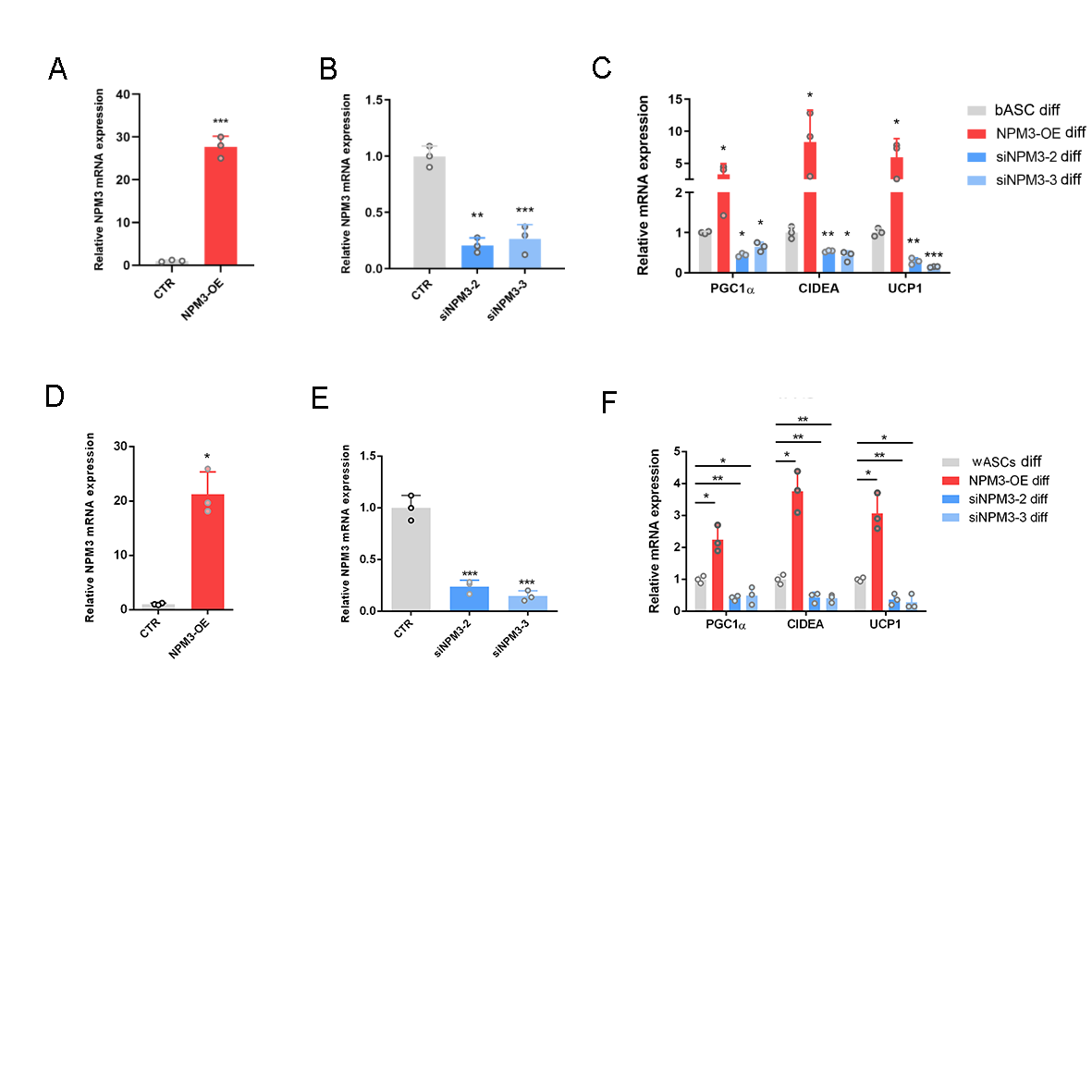


**Figure S4. Effects of NPM3 on the browning of ASCs from WAT and BAT.** Relative NPM3 mRNA levels after brown ASCs (bASCs) were infected with NPM3 overexpression lentiviruses (A) or transfected with NPM3 siRNA (B) and differentiated into adipocytes; (C) Relative mRNA expressions of PGC-1α, CIDEA and UCP1 at the late stage of bASCs differentiation (n=3); Relative NPM3 mRNA levels after white ASCs (wASCs) were infected with NPM3 overexpression lentiviruses (D) or transfected with NPM3 siRNA (E) and differentiated into adipocytes (n=3); (F) Relative mRNA expressions of PGC-1α, CIDEA and UCP1 at the late stage of wASC differentiation. (n=3). Data represent mean±SEM, *P<0.05, Student t test.


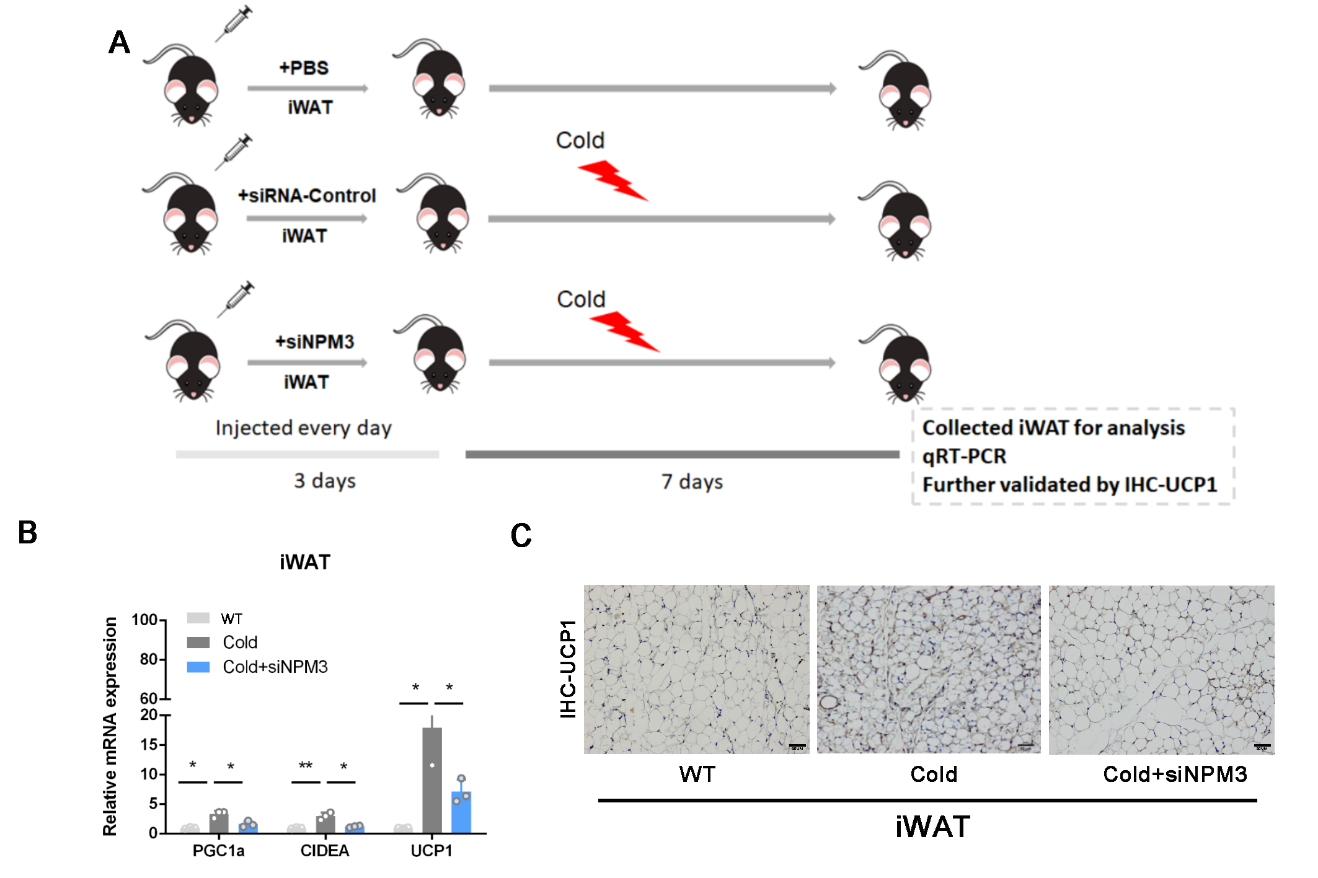


**Figure S5. Effects of NPM3 on cold-induced WAT browning.** (A) A flow chart depicting the in vivo knocking down NPM3 and browning induction in iWAT to confirm the functions of NPM3 in cold induced WAT browning; (B) Influences of NPM3 on the expressions of browning-related genes in iWAT after cold exposure (n=3), data were represented as mean±SEM and analyzed by student t test, *P<0.05, **P<0.01; (C) IHC staining of UCP1 in iWAT.


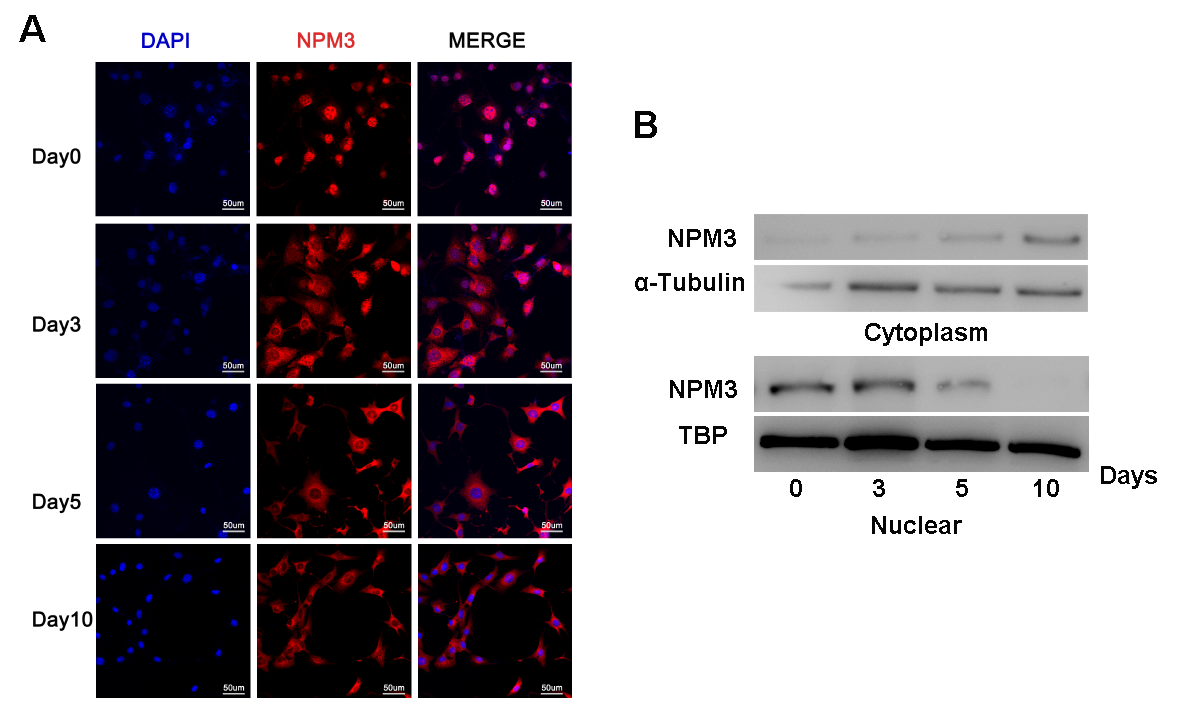


**Figure S6. The subcellular distribution of NPM3 in 3T3-L1cells during browning induction.** 3T3-L1 preadipocytes were induced for adipocytes for five days. Then the cells were treated with an induction medium supplemented with rosiglitazone for browning induction for another five days. (A)The subcellular distribution of NPM3 was detected by immunofluorescence (IF) on day 0,3,5,10 (scale bar=50μm); (B) Western blot was used to detect the expressions of NPM3 in the cytoplasm and nuclear of 3T3-L1 cells on day 0,3,5,10.


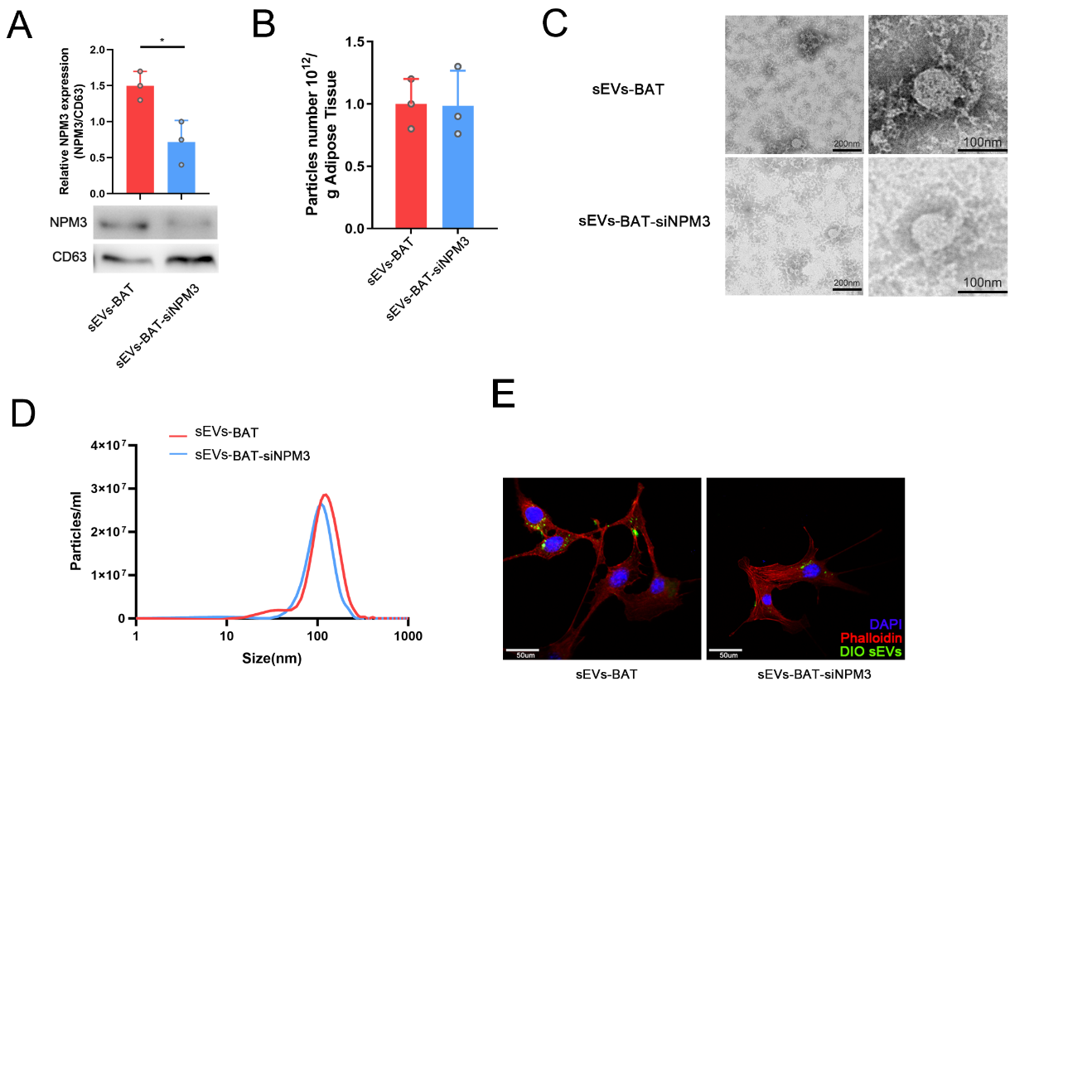


**Figure S7 Characterization of sEVs-BAT and sEVs-BAT-siNPM3.** (A) WB and semi-quantification analysis of NPM3 expression in sEVs-BAT and sEVs-BAT-siNPM3 (n=3), data were represented as mean±SEM and analyzed by student t test, **P<0.01; (B) sEVs-BAT and sEVs-BAT-siNPM3 release in BAT; (C) Electron micrograph of sEVs-BAT and sEVs-BAT-siNPM3, scale bar=200nm; (D) The size distribution of sEVs-BAT and sEVs-BAT-siNPM3 as determined by NTA; (E) 3T3-L1 cells were incubated with DiO-labeled sEVs-BAT and sEVs-BAT-siNPM3 (green) and stained with phallotoxins (red), nuclei were stained with DAPI (blue), scale bar=50μm.


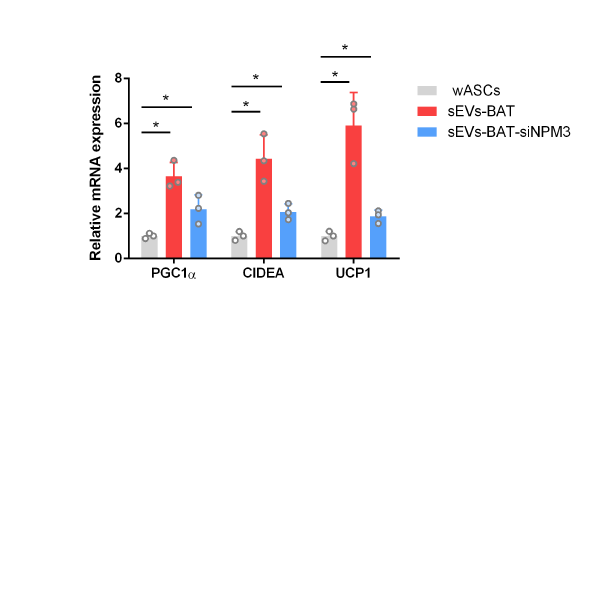


**Figure S8. Effects of** **sEVs-BAT and sEVs-BAT-siNPM3 on the browning induction of ASCs derived from white adipose tissue (wASC).** sEVs-BAT and sEVs-BAT-siNPM3 (50μg/ml) were co-cultured with wASCs for 10 days and relative mRNA expressions of PGC-1α, CIDEA and UCP1were detected by qRT-PCR, (n=3), data were represented as mean±SEM and analyzed by student t test, *P<0.05, **P<0.01.


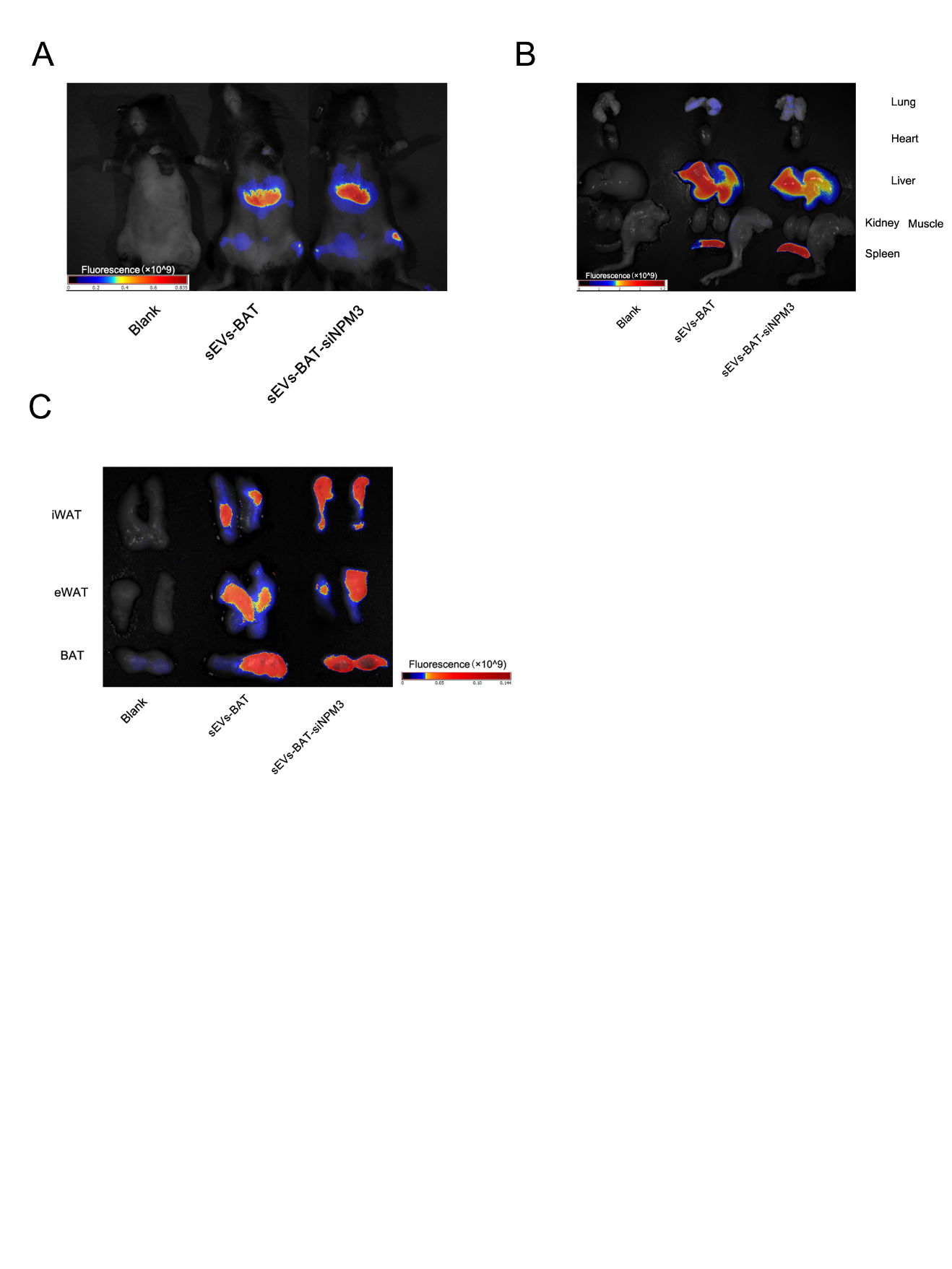


**Figure S9. Distribution of sEVs-BAT and sEVs-BAT-siNPM3 in vivo.** (A) Representative images of sEVs-BAT and sEVs-BAT-siNPM3 distribution in mice harvested at 6 hours following i.v. infusion of DiR labeled sEVs; (B) Representative images of different organs distribution (lung, heart, liver, spleen, muscle, kidney) 6 hours post-injection of DiR labeled sEVs-BAT and sEVs-BAT-siNPM3; (C) Representative images of adipose tissue (iWAT, eWAT, BAT) 6 hours post-injection of DiR labeled sEVs-BAT and sEVs-BAT-siNPM3.


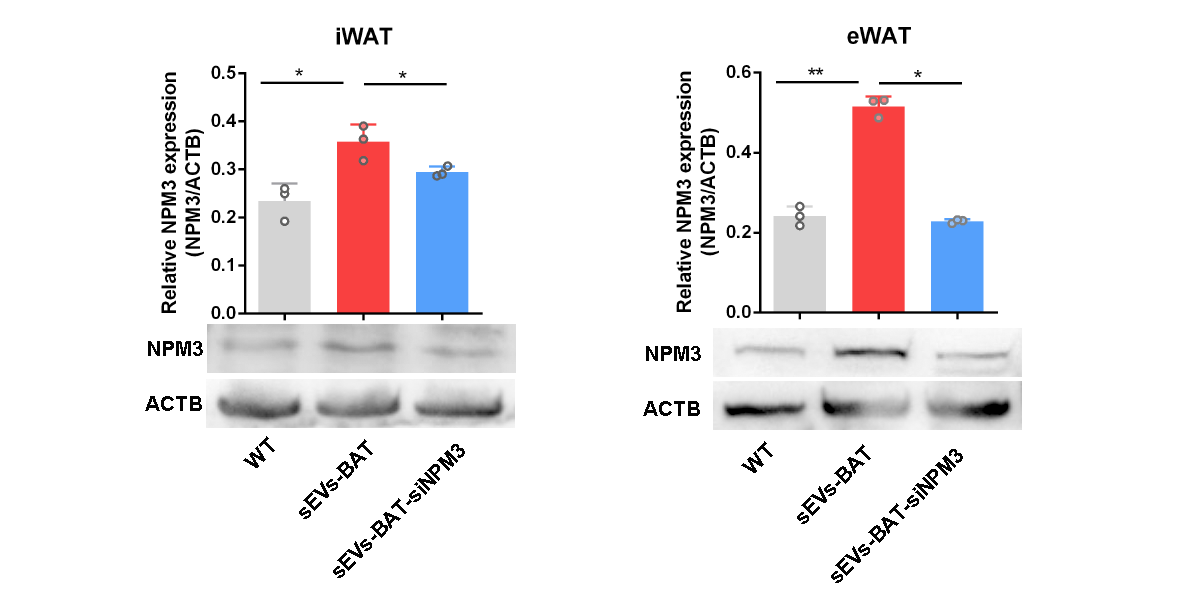


**Figure S10. Effects of sEVs-BAT or sEVs-BAT-siNPM3 on the expressions of NPM3 in iWAT and eWAT.** Mice were injected via the tail vein with sEVs-BAT or sEVs-BAT-siNPM3 (2μg sEVs/g body weight) every 2 days and lasted for 2 weeks. The expressions of NPM3 in iWAT (left) and eWAT (right) were detected by western blot (n=3), data were represented as mean±SEM and analyzed by one-way ANOVA followed by Tukey’s test, *P<0.05, **P<0.01, ***P<0.001.


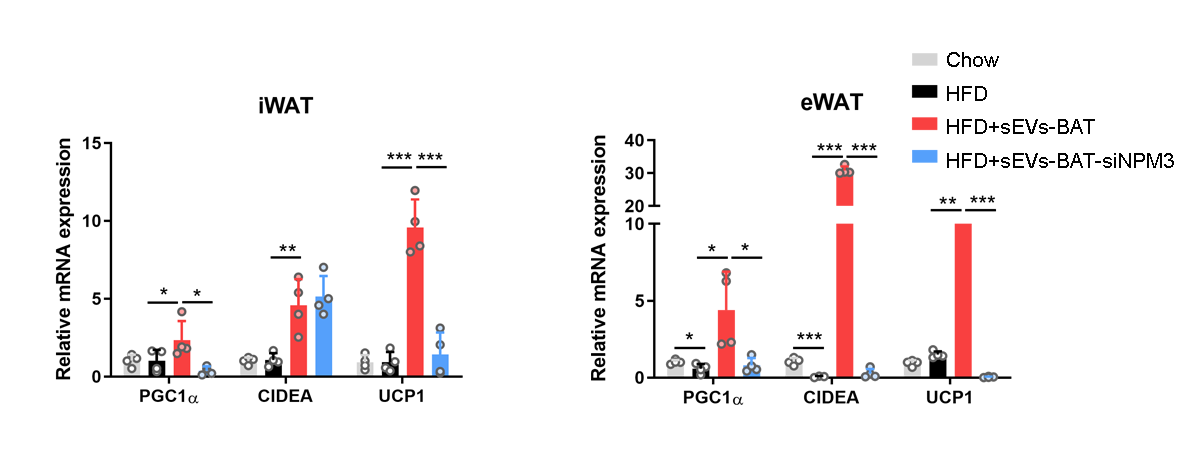


**Figure S11. Knocking down of NPM3 impaired sEVs-BAT mediated WAT browning in the HFD mice.** The mice were fed with a high-fat diet for 6 weeks firstly, then they were injected via the tail vein with sEVs-BAT or sEVs-BAT-siNPM3 (2μg sEVs/g body weight, n=6) isolated from the digested BAT every 2 days and lasted for 9 weeks. The expressions of browning related genes in iWAT and eWAT were detected by qRT-PCR (n=6), data were represented as mean±SEM and analyzed by one-way ANOVA followed by Tukey’s test, *P<0.05, **P<0.01, ***P<0.001.
