# Supplementary material for: Nucleophosmin3 carried by small extracellular vesicles contribute to white adipose tissue browning
Source: J Nanobiotechnology. 2022 Mar 28;20:165. doi: 10.1186/s12951-022-01381-1 (PMC8961928; doi:10.1186/s12951-022-01381-1)
Supplement: Supplementary file 2 — Additional file 2: Table S1. Antibodies. Table S2. Primers for qRT-PCR. [file 12951_2022_1381_MOESM2_ESM.docx]

**Additional file 2: Table S1. Antibodies.**

| Antibodies | Company | Catalog. No. | Dilution |
| --- | --- | --- | --- |
| Transferrin | Zen Bioscience, China | 251644 | 1:1000 |
| UCP-1 | Abcam, U.K. | ab23841 | 1:200 |
| ACTB | Abcam, U.K. | ab3280 | 1:1000 |
| NPM3 | BBI, China | D122780 | 1:1000 |
| IGFBP3 | BBI, China | D160507 | 1:1000 |
| SNRP70 | Santa Cruz, USA | sc-390899 | / |
| FLAG | CST, USA | 14793 | 1:1000 |
| CD63 | Zen Bioscience, China | 615509 | 1:1000 |
| TSG101 | Zen Bioscience, China | 341000 | 1:1000 |
| Apoa-1 | Zen Bioscience, China | 381145 | 1:1000 |

**Table S2. Primers for qRT-PCR.**

| Gene | Primers |
| --- | --- |
| EBF2 | 5’-CCTGAAGTGCAATCAGAATTGT-3’  GCACATGTCCATCCACATTTAC |
| PRDM16 | CAACAAAGAGAAGCCGTTCAAG  TTTCGGATCTCGGAGAAGTAAG |
| PPARγ | CCAAGAATACCAAAGTGCGATC  TCACAAGCATGAACTCCATAGT |
| HOXA5 | TAGTCACGACAATATAGGTGGC  GCATGAGCTATTTCGATCCTTC |
| PGC1α | GGATATACTTTACGCAGGTCGA  CGTCTGAGTTGGTATCTAGGTC |
| CIDEA | CAATGTCAAAGCCACGATGTAC  CTGTGCAGCATAGGACATAAAC |
| UCP1 | ATTCAGAGGCAAATCAGCTTTG  GTGTTTCTCTCCCTGAAGAGAA |
| aP2 | CATCCGGTCAGAGAGTACTTTT  TAGGGTTATGATGCTCTTCACC |
| IL-6 | CTCTGGGAAATCGTGGAAAT  CCAGTTTGGTAGCATCCATC |
| IL-1β | ATCTCGCAGCAGCACATCAA  ATGGGAACGTCACACACCAG |
| NPM3 | CAACTCCAACCACCTGTCACCTTC  GGCACCTGTAGCAAGCAGTTACC |
| ACTB | CTACCTCATGAAGATCCTGACC  CACAGCTTCTCTTTGATGTCAC |
| aP2 | CATCCGGTCAGAGAGTACTTTT  TAGGGTTATGATGCTCTTCACC |
| miR-133b | CGCGTTTGGTCCCCTTCAACCAGCTA |
| miR-455 | CGCGCTATGTGCCTTTGGACTACATCG |
| miR-133a | CCGTTTGGTCCCCTTCAACCAGCTG |
| miR-182 | GCCGCGTTTGGCAATGGTAGAACTCAC |
| miR-378 | CGCTCCTGACTCCAGGTCCTGTGT |
| miR-26b | CGCGCGCGCTTCAAGTAATTCAGGA |
| miR-193b | AACTGGCCCACAAAGTCCCGCT |
